# Exploring the working environment of Hospital Managers: a mixed methods study investigating stress, stereotypes, psychological safety and individual resilience

**DOI:** 10.1186/s12913-022-08812-7

**Published:** 2022-11-18

**Authors:** Kate Grailey, Clare Leon-Villapalos, Eleanor Murray, Stephen J Brett

**Affiliations:** 1 Department of Surgery and Cancer, Imperial College London, London, UK; 2 Imperial College NHS Trust, London, UK; 3 Said Business School, University of Oxford, Oxford, UK

**Keywords:** Psychological safety, Hospital managers, Resilience, Stress, Stereotypes

## Abstract

**Background:**

Hospital managers are responsible for the delivery of organisational strategy, development of clinical services and maintaining quality standards. There is limited research on hospital managers, in particular how stress manifests and impacts managers and the presence of individual resilience. Managers must work closely with clinical colleagues, however these relationships can be hindered by the perception of stereotyping and differing priorities. This study aimed to explore the working environment of hospital managers, focusing upon the unique stresses faced, psychological safety and the presence of resilience.

**Methods:**

This study utilised mixed methodology using an embedded approach. Participants were purposively recruited from all levels of hospital management within one National Health Service Trust in London, United Kingdom. An exploration of managers experiences was undertaken using semi-structured qualitative interviews. Psychological safety and individual resilience were additionally assessed using validated surveys. Qualitative data were analysed iteratively using inductive thematic analysis, and triangulated with quantitative data. Kruskal-Wallis statistical analysis was performed to evaluate differences in resilience and psychological safety according to seniority and background experience.

**Results:**

Twenty-two managers were recruited and interviewed, with 20 returning completed surveys. Key findings from the thematic analysis included the importance of good working relationships with clinical colleagues, the persistence of some stereotyping, and feeling unsupported in times of challenge. Stresses described included the bureaucracy involved when delivering change, conflict with colleagues and target driven expectations.

Participants described their own psychological safety as lower than desired, supported by quantitative data; but recognised its importance and strived to create it within their own teams. Sixteen participants had ‘normal’ scores for resilience, with senior managers more likely to have higher scores than those more junior (*p*=0.011).

**Conclusion:**

Positive working relationships, high psychological safety and individual resilience are important for organisational safety and individual wellbeing. Our data illustrate unique stressors faced by hospital managers, provide detail on sometimes challenging working relationships, and demonstrate scope to improve both the psychological safety and resilience of those in managerial positions. A map for senior healthcare leaders was constructed, facilitating the identification of modifiable areas within their organisation to promote good working relationships and improve the working environment of hospital managers.

**Supplementary Information:**

The online version contains supplementary material available at 10.1186/s12913-022-08812-7.

## Introduction

Hospital managers hold a significant amount of responsibility in key areas including oversight of large budgets and productivity, delivery of organisational strategy and priorities and the maintenance of safety and quality standards. Responsibilities also include ensuring patient safety, managing within financial constraints, delivering government targets and maintaining staff satisfaction [1]. To deliver on their priorities they must be effective communicators and strive to create a positive practice environment, both with managerial teams and clinical colleagues [2]. Relationships with clinical colleagues may be diminished by the persistence of stereotypes – with managers habitually viewed as being driven by financial goals [3, 4], rather than a focus on patient care.

There is limited research on how managers deal with stress, their perceptions of the working environment and interactions with clinical colleagues. Current research focuses upon first-line managers (those still possessing a clinical role [5–8]). Studies that do focus upon those in purely managerial roles are limited to questionnaires or surveys. One such survey conducted with senior health service staff found higher levels of stress, depression and anxiety than expected in senior managers [9]. A structured questionnaire in 67 managers found job stress to be significantly correlated with the presence of burnout [10].

Existing studies involving hospital managers are generally limited to two topics of investigation. Studies either explore the role of managers and their impact on patient safety and quality of care [1] or investigate their competence to perform the role (both self-perceived [11, 12], whether they have the skills required for the role (such as communication and financial awareness [2]), or their impact on employees [13]).

Individual, team and organisational factors can impact on the stresses faced by those in managerial positions and their ability to perform their role. These include psychological safety as an individual and team construct, and resilience as an individual attribute.

Psychological safety can be defined as an “environment safe for interpersonal risk taking” [14], and its presence in clinical healthcare workers has been explored previously by this research group [15, 16]. The presence of psychological safety in hospital managers has not been quantified in the academic literature, despite it being likely important that they feel psychologically safe when interacting with those in more senior positions (facilitating the raising of concerns and proposed innovations [14, 17]). It is also important that those occupying management positions foster a culture of psychological safety within their own teams and those reporting to them [18].

Resilience, defined as the ability to “bounce back” [19] and positively adjust to adversity [20] is a key attribute in National Health Service (NHS) leaders [21] and staff [22]. Resilience assists individuals in leadership positions to support their team, manage difficult situations and protect their own wellbeing [23]. There are limited studies looking at the presence of resilience in healthcare managers (despite it being frequently studied in healthcare workers themselves). Those studies that did focus on managers investigated how managers create resilience within their staff, rather than exploring their own resilience [24].

This study was one of the first pieces of research exploring the experiences of hospital managers in their workplace. As such, the aims were deliberately broad. Our intention was to explore a number of facets that we anticipated would be important in a manager’s working environment, and that may contribute to stress. There were 4 research aims designed to explore the working environment of those in management positions:

- To investigate how hospital managers perceive their role within the working environment, including:
  - ◦ An exploration of working relationships and the perception of stereotyping.
  - ◦ Investigating the impact of a participant’s background.
- To explore how psychological safety manifests, namely:
  - ◦ How psychologically safe to hospital managers perceive themselves to be?
  - ◦ How do they foster a culture of psychological safety in the teams they manage?
  - ◦ Are there negative consequences associated with a psychologically safe environment?
- To explore and quantify the presence of individual resilience in our sample of hospital managers.
- To explore the stresses faced by hospital managers within their workplace and any contributory factors.

## Methods

Study protocols were reviewed and approved by the Imperial College Research Governance and Integrity Team (Reference Number: 20HH6472), the Health Research Authority (REC Reference 21/HRA/0013) and the NHS Trust studied. Data collection took place between February 2021 and August 2021.

### Study setting

The NHS Trust studied is a large NHS trust in London, serving approximately 1 million people and employing 14,500 staff members. The trust comprises five hospitals, three of which provide acute and specialist services and were selected as sites for participant recruitment. An understanding of the structure of the management team within the NHS Trust studied was obtained through prior discussion with senior managers and publicly available data on managerial positions within the trust. Within the NHS there are several groups of hospital managers with differing responsibilities, including clinical, financial, human resources, estates, administrative, general and operational managers [25]. This study focused upon those working in a general management role, as the responsibilities of these individuals can include service delivery, staffing and multi-million-pound budgets. For those in general managerial roles, these can range from more junior support roles such as service coordinators, with a focus on operational delivery, through to hospital managers and divisional directors, whose roles encompass oversight and delivery of strategic priorities within the trust.

### Participant recruitment

Recruitment to the study was purposive, ensuring a wide range of managerial levels and viewpoints were present. Participants were invited to participate in the study if they held a general managerial position ), regardless of seniority, specialty or division.

### Data collection

To address the research aims, an embedded approach to mixed methodology was designed, with qualitative interviews supported by quantitative survey data. Interview topic guides were designed based upon existing literature and previous research on psychological safety and teamwork in the healthcare environment by this research group [15, 16]. The topic guide included questions relating to the participant’s perception of their working environment, their psychological safety and the influence of their working background. COVID-19 social distancing restrictions were in place during the study therefore qualitative interviews were undertaken virtually via the Microsoft Teams platform. Participants provided written informed consent prior to the interviews and were free to leave the study at any time. Interviews were conducted by one researcher within the team (KG).

Following each interview participants were asked to complete a short 13-part survey. This comprised the 6-part Brief Resilience Scale (BRS) [26] and a scale containing 7 statements used to evaluate the presence of psychological safety [14]. The BRS is designed to evaluate the presence of resilience by measuring the ability of an individual to “bounce back” or adapt to stress. It has been shown to have good internal consistency and test re-test reliability [26]. The scale used to assess psychological safety was developed by Edmondson [14] during her study of real work teams (built from work regarding team shared beliefs, learning behaviours and performance). This scale has been adopted widely in the investigation of psychological safety [27, 28], and has demonstrated high internal consistency reliability and discriminant validity [14]. To our knowledge, neither scale have previously been used in a sample of hospital managers.

The initial interview topic guide and surveys can be viewed in Supplementary File 1.

Interviews were recorded and transcribed with all personal identifying information removed. Qualitative interview transcripts were analysed alongside ongoing data collection as an iterative process. This was primarily conducted by one researcher (KG), with ongoing discussion and input from the research team. This informed the direction of future interviews, allowed exploration of novel topics and an assessment of thematic saturation [29] (defined as the point at which no new codes are added to the thematic framework). Recruitment ceased at the point of thematic saturation.

### Data analysis

A thematic analysis approach was applied to qualitative data. This was performed primarily by one researcher (KG), using NVIVO R1 (QSR International) to facilitate data handling. The thematic analysis was inductive with themes arising from the data, rather than being driven by pre-existing ideas or frameworks. The process of thematic analysis followed an acknowledged sequence [30], including familiarisation with the data set, review and generation of initial codes, re-review of data for themes and the construction of a thematic framework. Throughout each stage data were re-reviewed in a recursive process, and the resulting analysis discussed with the wider research team. 10% of the transcripts were selected at random and coded by a second researcher (CLV) to assess for inter-rater reliability, using a coding comparison query within NVIVO R1.

Quantitative data were analysed within Microsoft Excel and IBM SPSS v28 to generate descriptive statistics regarding the presence of resilience and psychological safety within our participant group. There were four participant subgroups – those with a clinical versus non-clinical background and junior versus senior managers. A Kruskal-Wallis statistical analysis was performed on summed Likert Scale data to assess for any differences in the presence of psychological safety or resilience between these sub-groups.

### Reflexivity

For reflexivity, KG is a postgraduate researcher with a background in anaesthesia and critical care, CLV is a lead Nurse for Practice Development and Innovation. EJM is a former NHS manager who is now an academic in organisational studies and SJB is a clinical academic and consultant in intensive care. All have previous experience with the conduct and analysis of qualitative studies in a clinical environment. The authors were aware of how their own position may affect the study design, analysis and interpretation of the findings and maintained a reflexive position throughout the analysis to minimise the risk that any presumptions would affect the analysis and interpretation of the study findings.

This manuscript is written in accordance with the Standards for Reporting Qualitative Research (SRQR) checklist [31] (Supplementary File 2).

## Results

Twenty-two managers were recruited and interviewed, with 20 returning a completed survey. In line with purposive sampling and the inclusion criteria a broad range of managerial roles were recruited. The participant group were subsequently organised into those more junior (individuals whose role typically had a closer focus upon operational delivery of their service), and those more senior (individuals typically responsible for operational delivery, multiple services and the strategic delivery of trust priorities). This allowed further evaluation and comparison of resilience and psychological safety across these two groups. The profile of the participant group can be viewed in Table 1.

**Table 1:** Profile of participant group

Level of Seniority	Managerial Level / Job Description	Background	Number of Participants
Junior	Service Support Manager	Non-Clinical	2
	Service Coordinator	Clinical	1
	Deputy General Manager	Non-Clinical	1
	Business Manager	Non-Clinical	2
Senior	General Manager	Clinical	5
		Non-Clinical	5
	Operations Manager	Non-Clinical	1
	Programme Director	Non-Clinical	1
	Divisional Director	Clinical	1
	Hospital Director	Clinical	1

Interviews ranged between 24 and 48 min, with an average length of 31 min. Thematic saturation was achieved within the first 18 interviews, with only minor modifications made to the coding framework after this point.

Sixty-nine codes were creating during the thematic analysis. Cross-coding revealed that the percentage agreement was > 96.3% across all codes, with 19 codes having a kappa coefficient of > 0.75, and a further 4 having a kappa coefficient between 0.4 and 0.75. Any discrepancies were resolved through discussion between the study team.

**Fig. 1:**
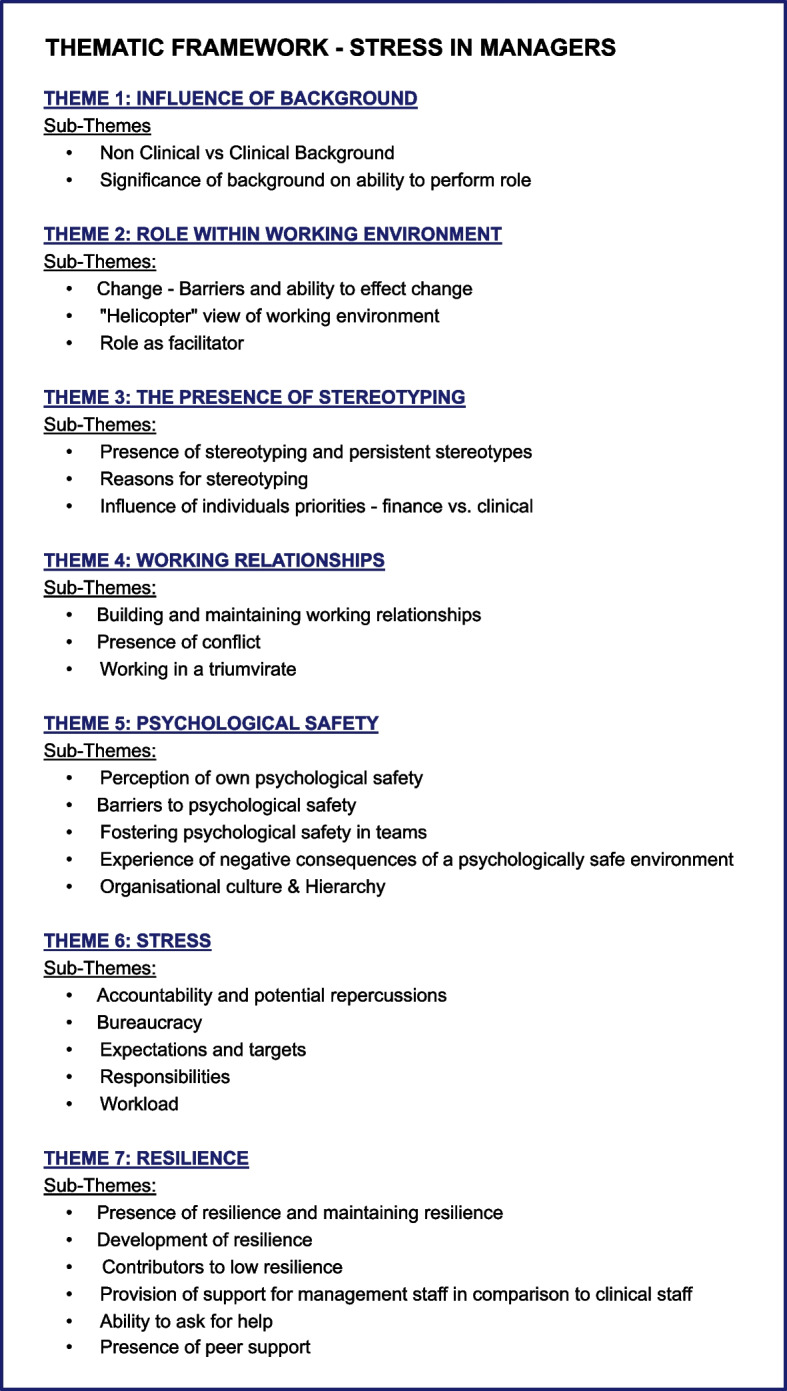
Thematic Framework outlining the themes and sub-themes constructed during the thematic analysis

The thematic analysis yielded a wealth of data regarding hospital managers’ perception of their working environment, resilience, psychological safety and stress. A thematic framework was constructed housing 7 main themes (Fig. 1).

Four themes related to the first research aim - manager’s perception of their workplace, with the remaining three themes corresponding to the research aims of investigating psychological safety, resilience and stress.

We present the qualitative results of the thematic analysis according to the constructed framework, organised as per the research aims. The quantitative data relating to psychological safety and resilience are embedded within the corresponding qualitative themes. Further supporting qualitative data is presented in Supplementary File 2.

### Managers perceptions of their working environment

#### Theme 1: influence of background

Seven participants had clinical backgrounds, with the remaining 15 having either managerial / business backgrounds, or had progressed through the NHS in administrative pathways. Data within this theme indicated that participants viewed themselves as a heterogenous group, with variable skills, backgrounds and abilities.

Many participants described differences in those who had had a clinical background, contrasting those with managerial/business backgrounds. In general a clinical background was perceived to result in better understanding of the working environment and more credible communication with clinical staff. It was also acknowledged that having a non-clinical background brought different benefits and insights to the role. These included analytical ability, objectivity and having dedicated training in specific managerial skills.

“I think so because I think it’s really good that they do have it [*a clinical background*]. They have a better understanding of the patients, and the patient’s needs and what you are up against with your theatre lists as an example. *0114, Service Coordinator*

if they know what the procedures are. Yes, just even understanding the patients, particularly MDTs [*this means multi-disciplinary teams*] and that sort of thing. I think it’s good to know.” *0114, Service Coordinator*

“I’d like to believe it [*a managerial background*] allows me to have an unbiased view. It’s not formed based on my own personal experiences of working within clinical areas. The flipside of that is really I have to rely on experts and try to interpret whether the experts are feeding me their priorities or the priorities for the specialities throughout the organisation.” *0101, Hospital Director*

It was also conveyed that working collaboratively with a heterogeneous group of managers possessing different skills sets was beneficial for service delivery.

“And a manager who comes in as manager will be much more focused on maybe various outcomes but not necessarily on how to resolve things and get there in a way that works for the team or they’ll do it in a different way. But I think strengths of the NHS is the variety of managers that you’ve got.” *0107, General Manager*

#### Theme 2: role within working environment

Participants viewed their role within the working environment in several ways. These views incorporated their ability to enact their role and responsibilities, and their freedom to innovate and use their initiative. A prominent topic was participants’ ability to identify areas for change, and barriers to enacting such change. Generally, participants were positive about their ability to do this, particularly within their own directorate, however they identified the importance of engaging stakeholders, both managerial and clinical.

*“*I think most of the time if you get the right people in the room, and they don’t feel done to, that they’re part of it, it, sort of, normally works.” *0095, General Manager*

Participants described possessing a “helicopter” view of the working environment, based on their viewpoints, understanding and working responsibilities covering several clinical areas – typically those in more senior managerial roles had oversight and responsibility for several clinical areas and services. This was described as allowing them to have a greater understanding of how several services interacted and shared budgets than their clinical colleagues.

*“*And I think that’s, in some ways, you can’t criticise for them for that because they’re only seeing the bit they see… And our job is to have that overarching responsibility and accountability, but sometimes it’s a bit difficult to explain that maybe you’re not the priority” *0095, General Manager*

Often managers described themselves being in a facilitatory role – bridging the gaps between stakeholders, explaining reasons for delays and providing clinical staff with the resources they need to care for patients. This faciliatory role was particularly prominent when trying to secure funding for new equipment or resources.

*“*I see my role as being able to facilitate some resourcing that they need to be able to do their job, that’s a very big part of my role” *0116, General Manager*

*“*I think, upfront I usually say, look, my deadline is X, and probably yours is Y, but if we can try and meet in the middle and understand where I’m coming from, so explain to them why because I wouldn’t just have a deadline for a deadline’s sake” *0104, General Manager*

#### Theme 3: perception of stereotyping

The exploration of stereotyping within the manager-clinician relationship led to three subthemes – the perception of stereotyping and what the reported enduring stereotypes were, the reasons behind this perceived stereotyping and the influence of individual priorities.

There was a heterogeneous response when participants were asked about their perceptions regarding the presence of stereotyping. Many felt that it was “outdated”, and did not feature in their day-to-day working life. These perspectives contrasted with persistent views in some participants that clinicians often saw managers as purely finance- or target-focused, and that this stereotyping affected their working interactions. Participants highlighted that for their own development they often rotate through roles as they progress in seniority. As such, they perceived that clinicians often viewed this negatively – expecting managers to not be in each post for long, and thereby discrediting their views.

“So, clinicians will think, oh, this is about, to make them all balancing the books. Whereas, actually, it’s a real opportunity to set out what you want. Any business plan is about what you want to do. What you want to do on behalf of your patients and your people, and what that means to your bottom line, in terms of money.” *0108, Divisional Director*

“So, we worked quite closely alongside each other since the day I got here, and one of the first things he said to me was, management never stay. This is a consultant-led kind of environment because management just don’t stick around. If I don’t get on with management, I’ll wait until they disappear, and the next lot to come in in 18 months’ time and we’ll just carry on that way.” *0096, General Manager*

Where stereotyping was perceived by participants to be present, it was described as being driven by a clinician’s lack of insight regarding the responsibilities of a manager and the constraints they faced. This echoes the previous sub-theme of managers having a “helicopter” view of the workplace environment, and a lack of understanding regarding managerial roles and responsibilities. It was also felt that clinicians didn’t understand that managers also viewed improving patient care as a priority in their role but delivered this via different mechanisms, such as resource allocation. The ongoing persistence of stereotyping was additionally perceived to be influenced by the different priorities of individual stakeholders, classically clinical vs. financial.

*“*But I think that’s people not understanding we’re responsible for the governance, we’re responsible for the quality and safety. It’s a lack of understanding.” *0102, Deputy General Manager*

“So, I think, yes, that we do come across it a lot. And, you almost have to make more of a deal of it, because I think people expect you just to be interested in money. But of course, if you look after the money, we can provide care for more patients. So, yes, actually I’m worried about all the patients.” *0106, General Manager*

“I think the consultants are more interested in the patient, absolutely and they don’t want to learn about finance but they recognise that finance is there, but they expect somebody else to deal with that.” *0107, General Manager*

#### Theme 4: working relationships

Building and maintaining relationships with clinical colleagues was clearly very important to our group of participants, with building good working relationships seen as being essential to success (in terms of service delivery, patient care and generating lasting improvement).

*“*Managers come and go, but the clinicians tend to be there for a long time, and it’s their service and they’re the ones that are usually really passionate about it. And you really have to build your relationships with them in order to really drive through the changes and the improvements that you want.” *0117, Service Coordinator*

Incidents of conflict and disagreement between clinical staff and those in management positions was frequently described by participants, generally underpinned by tensions around limited available financial resources and subsequent limitations on service improvements. This conflict took the form of both verbal and written communications between staff. It was also felt that managers bore the brunt of clinicians’ frustrations when requests weren’t approved, even though this was out of their control.

*“*Sometimes you get consultants or nurses who, sometimes due to an experience or sometimes due to frustration, feel that it’s the wrong decision. And clearly trying to manage that reaction is sometimes tricky. But I think to a certain extent it goes with the turf, unfortunately.” *0101, Hospital Director*

Many of the mangers interviewed worked in a triumvirate model – comprised of a senior nurse, senior doctor and manager. This model allowed representation of different views and priorities when key decisions were being taken. This was reported as being a beneficial way of working as the combination of skills and experiences worked in synergy.

*“*I think the triumvirate works very well if you get one doctor, one nurse, and one manager, I think, because you all bring different perspectives towards the same goal. And I think if the triumvirate works well in cohesion, I think the success can be really, really incredible.” *0109, General Manager*

##### Theme 5: Psychological Safety

There were two main focuses regarding psychological safety during the interviews – the perception of a participant’s own psychological safety and how they created this environment within their own team.

Participants conveyed mixed views when discussing their own psychological safety, with a significant number of negative factors present that diminished participant’s perception of their own psychological safety. Factors such as experience within the role and knowing other team members were described as facilitating psychological safety, particularly related to speaking up behaviours. Many participants expressed that they felt “safe” to speak up, but had little faith that their concerns would be acted upon or would lead to change. There were examples of managers being concerned to raise things to those more senior within the organisation, subsequently utilising alternative routes to raise concerns.

“I think it depends which executive you’re talking to. If I wanted to raise a concern, yes. if I wanted something done about said concern, that responsibility firmly sits at my feet.” *0105, General Manager*

Barriers to an individual’s psychological safety included fear of repercussions, risking negative reactions from seniors, a lack of availability/opportunity and difficulty being heard within large meetings. These concerns were reflected in a discussion regarding the organisational culture and how it impacted on psychological safety. Some participants described organisational cultures that discouraged speaking up, contrasting with other participant views that a cultural shift was developing to improve psychological safety.

“I don’t think that there’s a strong culture of standing up and saying this isn’t right or I don’t agree with it or is there a better way of doing it.” *0098, General Manager*

Interestingly, in contrast with participants’ impression of their own psychological safety, they were almost universal in positively describing the psychological safety of their own team. This was reflected in both the way psychological safety was created, and descriptions of its manifestation. Participants felt they were good at fostering an environment of psychological safety, creating an “open door policy”. A very strong ethos of participants feeling that their team could feel able to come and speak to them about any issue was present within the data.

“I personally do and particularly encourage my team, the team a level below me and a level below that to do the same, to get the feedback and get, how can we change things, how can we do things? It’s not always easy.” *0107, General Manager*

Some negative aspects to a psychologically safe environment were present within the data, that arose as a consequence of fostering such an environment. These included having their time taken up with multiple concerns and queries, knowing when to intervene in issues raised, and also feeling that they were responsible / accountable for problems once raised. These perspectives were particularly prevalent in those within the study sample in more senior roles.

“Yes, it has quite a few downsides. There’s a purely personal one, which that it can be really exhausting. So if you set yourself as a “I’m going to be someone who anyone can speak to”, you have to be willing to accept the fact that anyone might come and speak to you. As well as taking up a lot of time, which is fine, you can manage that, it can be really difficult to absorb all those other issues.” *0100, Divisional Director*

**Fig. 2:**
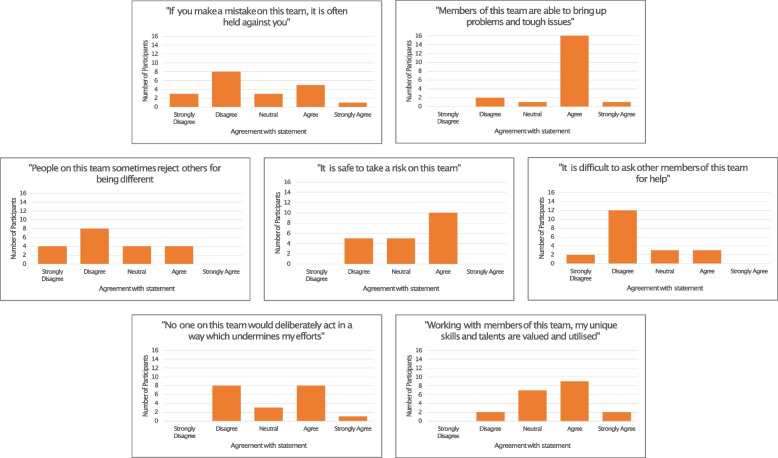
Participant agreement with statements assessing the presence of a psychologically safe environment

The heterogeneous nature of the qualitative data on psychological safety in the participant group was reflected in the quantitative survey data (provided for 20 participants) (Fig. 2). 30% of participants (n = 6) agreed / strongly agreed that if they “made a mistake on this team it would be held against them”, 40% of participants (n = 8) disagreed that “no one on this team would deliberately act in a way which undermines my efforts”, 25% (n = 5) disagreed that they felt “safe to take a risk on this team” and 20% (n = 4) agreed that “people on this team sometimes reject others for being different”.

A Kruskal-Wallis test [32] was performed on the summed means from these Likert items, comparing the psychological safety of those with a clinical background vs. a non-clinical background, and according to level of seniority (defined as junior vs. senior and illustrated in Table 1). Participants who had a clinical background demonstrated higher perceived levels of psychological safety, although this did not quite reach statistical significance (H(1) = 3.73, *p* = 0.053). There was no significant difference in psychological safety according to level of seniority (H(1) = 0.148, *p* = 0.7).

#### Theme 6: resilience

Qualitative data regarding the presence of individual resilience, contributors to low resilience, and how resilience was developed were obtained, leading to an iterative broader discussion regarding the availability of support for managers.

Many participants confidently stated that they perceived themselves to be resilient, and provided examples of previous experiences that had contributed to this. Such experiences included successfully managing the challenges associated with their working role, dealing with conflict and learning from experience.

“I think, I don’t know, it depends on how resilient you are, but I’ve learned resilience because you know that the things you might be stressed about one day, probably aren’t going to be the things you’re stressed about the next day and the next day.” *0106, General Manager*

Participants also discussed what contributed to low resilience, including frustration with financial constraints, working with difficult colleagues and lack of support.

“I think there’s a bit about working with difficult people as well. I think as you work higher up or climb the ladder or whatever you call it, you do face a lot more challenges in getting things approved, getting things done, and you do face a lot more obstruction” *0110, Business Manager*

The theme of managerial staff lacking support (particularly in comparison to their clinical colleagues) was prominent within the qualitative data. Managers reported feeling unsupported, unappreciated and without provision of a pathway to support them if needed. These perceptions reflected a perceived lack of support both within local teams, and the wider organisation as a whole.

“And like you said, we’re forgotten as admin I would say. That there’s this whole work we’re doing in the background, but nobody really thinks that we still have to come to work every day. We have to do all of that. We still have a personal life and problems in personal life…….. So, I think we are a little bit overlooked in how things can be done.” *0112 Service support manager*

The iterative nature of the topic guide, and the strength of participant feelings regarding their lack of support subsequently led to participants being asked whether they felt able to vocalise if they were struggling or needed extra help. Many stated that this was not the culture within organisations and that they were expected to “just get on with it”.

“Oh, absolutely. You can’t admit that you’re struggling. You have to do long hours. It’s accepted of you. You can’t possibly leave at five o’ clock. And, definitely, most people are martyrs, of course.” *0105, General Manager*

*“Yes, I think so, definitely, particularly the more senior you get in an organisation, it’s seen as a sign of, kind of, weakness, which is wrong because basically I think sometimes when you talk over something with another colleague or a secretary, you can work it out much quicker. I think managers are seen to be a different breed, and actually we’re not” 0104, General Manager*

Whilst formal organisational support was universally described as lacking by participants, there was a sense within the data that peer support from other managers had improved recently. This was particularly prevalent in the group of general managers, having experienced training courses together that led to increased collaboration on future work. This increased collaboration across directorates had the effect of enhancing group psychological safety and improving problem solving.

*“has really brought us much closer together, and across divisions, across directorates, whereas before we tended to work really quite siloed. But now I think, yes, definitely. I know somebody who’s got skills in another area who I would have no problem going up to and saying, look, basically, I know you’ve got the experience with this, how did you work this out, and vice-a-versa. People will come to me. So, yes, to share skills and knowledge is good.” 0104, General Manager*

*“So, you really need your peers, and I think, and it must be the same in medicine as well. It’s just talking, oh, and everyone’s got the same issues. You can often sort problems out. When I was a deputy manager as well, the camaraderie you get, I think, is really important. That, almost more than anything, is a good way to just take the overwhelmingness of it.” 0106, General Manager*

*“if you get all of us together, there’s actually not a lot that we can’t do. And there’s not a lot of people that can say we can’t do it, either, because we’re all together.” 0094, General Manager*

The qualitative analysis demonstrated that participants believed themselves to have become resilient due to prior experience of organisational challenges. One individual had low resilience (values < 2.99), 16 scored within the normal range for resilience (3.0-4.3) and 3 participants had high resilience (> 4.31).

**Fig. 3:**
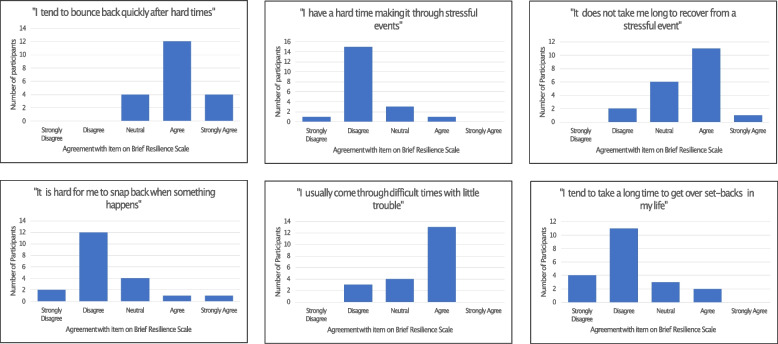
Participant agreement with the 6 statements comprising the Brief Resilience Scale

These scores, with a majority of individuals scoring “normal” for resilience are reflected in the breakdown of the answers for each statement, illustrated in Fig. 3.

A Kruskal-Wallis test [32] was performed on the summed Likert Scale data to ascertain whether a participant’s background or level of seniority influenced the resilience they had within the workplace. Those more senior were statistically more likely to have higher scores for resilience (H(1) = 6.445, *p* = 0.011). There was no significant difference in resilience according to a participant’s background (H(1) = 0.518, *p* = 0.472).

##### Presence of workplace stressors

A major stressor identified by participants was the pressure to meet targets, and their own accountability if these targets were not met. In addition, the amount of bureaucracy navigated when trying to implement changes was highlighted as a frequent source of stress.

*I think the most stressful bit is having to sit in front of a panel of people and explain why you weren’t able to deliver the impossible.” 0110, Business Manager*

“Even though we work as equals, at the end of the day, if anything goes wrong, then all the joy of the accountability sits with me.” *0116, General Manager*

“I think there is far more bureaucracy in the NHS that impacts on what we do. So, the red tape to get things done and the delays that are out in about decision making really makes the job hard.” *0107, General Manager*

Stress caused by the expectations and targets placed upon managers was a prominent sub-theme, exacerbated by the fact participants often stated that meeting these targets was difficult and was out of their control. Participants reported having significant responsibilities – particularly in relation to the size of the budgets they were in control of, as well as the stress of ensuring the decisions they made around resourcing patient care were the right ones.

“So, if you’re looking at individuals who have to manage businesses that turn over £50 million a year that’s a big-time business, and that’s a big-time team, but that’s similar to what each General Manager at [Company] do. And it’s like a big-time business. You’ve got all of your huge expensive equipment, you’ve got all your expensive clinicians, you’ve got your safety metrics, quality metrics, financial accountability. That’s a full business unit within a wider hospital” *0113, General Manager*

“And I still feel quite stressed by the responsibility of that and making sure they’re okay. Even though I know it was the right thing to do and I stand by the decisions I made” *0103, Programme Director*

These stressors all fed into the final subtheme – which related to workload (and by default work life balance), with many participants reporting that they often worked significantly beyond their scheduled hours. This was influenced both by volume of work but also an expectation from clinical staff that they would be available at all times.

“because unfortunately everyone comes to you, but you don’t have anyone to go to. And then it gets to the point where you’re not sleeping. Managers don’t get paid over time. I would be going in on the weekend. You don’t get paid for that. That’s my decision to go in.” *0102, Deputy General Manager*

### Key factors identified throughout data analysis

**Fig. 4:**
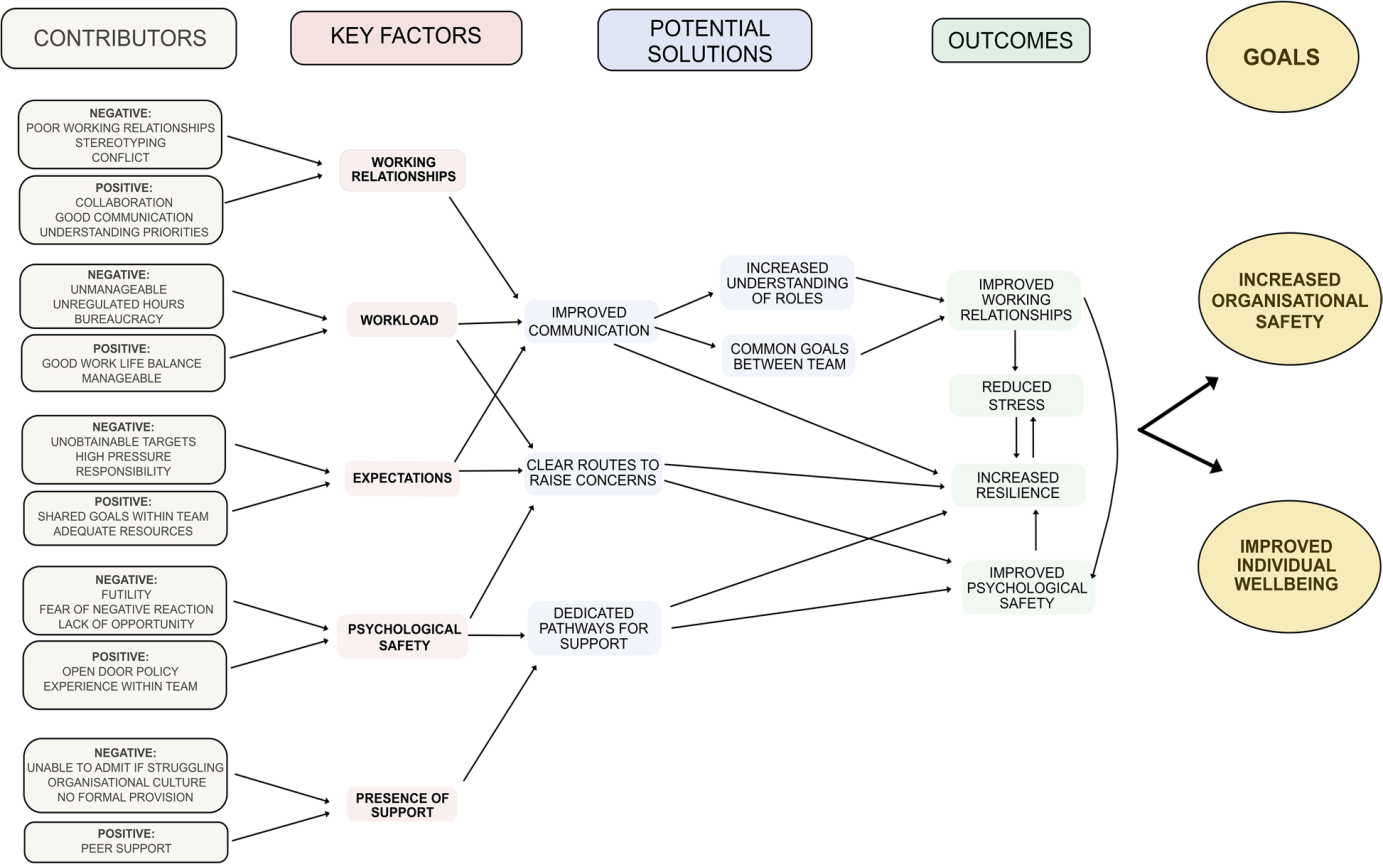
A map highlighting positive and negative contributors to factors that influence the goals of organisational safety and individual wellbeing in hospital managers

The mixed methods analysis allowed contributing themes and key factors influencing an individual’s wellbeing and the level of organisational safety to be identified. Data were re-reviewed, dividing these themes according to whether they had a positive or negative influence. This information was utilised to develop solutions that might improve outcomes such as psychological safety and subsequently the goals of improved individual wellbeing and organisational safety. These ideas and themes were used to construct a map, highlighting potential points for modification and how these factors may be inter-dependent upon each other (Fig. 4). The aim of constructing this map was to create a tool for senior healthcare leaders and managers that would allow them to identify the presence of these key issues within their own workplace. It is envisioned that this map will assist in the design of interventions to improve working conditions for hospital managers and the wider healthcare leadership team.

## Discussion

This study yielded a wealth of novel information regarding the behaviours and experiences of hospital managers within the NHS, particularly with respect to the manifestation of stress and psychological safety; most had worked in numerous NHS organisations and therefore provided evidence reflecting wide experience. These insights are useful for those wanting to mitigate some of the stress felt by managers, improve their working conditions and develop better working relationships between managers and clinical staff.

The perception of stereotyping (and subsequent expectations of how individuals will behave in the workplace, particularly with respect to their work priorities) is well acknowledged within the existing literature [3, 4, 33, 34]. The data in this study provide insights into how this stereotyping can manifest - driven by a lack of understanding of each other’s roles and different targets and priorities. Positively, the analysis also demonstrated a clear trend in the dissipation of such stereotypes – described by many participants as outdated.

It was interesting to note the acknowledgement by many managers that clinicians disliked them moving positions frequently. This may reflect lack of insight into career pathways, specifically that junior managers should and must move between departments and organisations to gain experience and seniority. As such junior managers could be considered as “in training” as well as responsible for role delivery.

Despite the ongoing perception of stereotypes, all agreed that developing and maintaining good working relationships was key when implementing change and service improvements. A prominent theme within the qualitative data was that whilst a manager’s role was primarily finance or target focused, this sits within the overarching aim of delivery of high-quality healthcare to patients. Explicit acknowledgement of shared strategic goals should build clinician – manager relationships.

This study aimed to explore the perceived impact of an individual’s background on their ability to act in a managerial role. Many participants acknowledged that there were differences in managers with a clinical background vs. those employed via other routes. Generally there was a view that when taken as a collective, these differing backgrounds provided benefits, including improved peer support and collaboration. Interestingly, managers with a clinical background demonstrated higher scores for psychological safety, perhaps reflecting a longer period of time spent in the healthcare workplace, or a greater understanding of how and when to speak up in this environment. This may also reflect an expectation for ongoing reflective practice in those working within the healthcare setting.

The manifestation of psychological safety was explored in two different ways within the interviews. When asked about their own psychological safety, views tended to reflect a more “unsafe” environment. More negative perceptions of a participant’s own psychological safety were predominant in more senior managers, perhaps reflecting the seniority of those to whom they were required to raise concerns to within the organisation. A perception of being unable to speak freely and raise concerns may have grave implications for organisational safety and learning. This highlights a potential focus for future exploration into the flow of information between those at different levels within the management structure of complex organisations.

Despite describing their own psychological safety as sub-optimal, all participants agreed that they were able to create an open culture, encouraging speaking up and raising ideas in the teams they managed. It would be interesting to evaluate this discrepancy in perceptions regarding psychological safety – to ascertain if it is only more senior managers who lack psychological safety, or if their belief that their team is psychologically safe is not corroborated by those more junior.

Additionally, the conversation regarding psychological safety elicited detail regarding the potential negative consequences of psychological safety, particularly for the team leader, which is another topic that warrants further exploration.

Specific stressors for those in management roles included the presence of bureaucracy when trying to design and implement change and the expectations placed upon managers to deliver, particularly focused upon the volume of paperwork and approvals required for new initiatives.

Several approaches to the development of individual resilience are described in the literature. The psychology literature increasingly recognises the dynamic nature of resilience over an individual’s lifetime as a response to challenging situations [35, 36]. Applied literatures recognise the role of interventions or techniques designed to teach and promote resilience e.g. facilitating controlled decision making or focusing upon building strong relationships within the workplace [37–39]. In this study, participants discussed how they had developed resilience as a function of repeated negative experiences and stress, and what contributed to low resilience. Whilst the majority of participants scored “normal” for resilience, only 3 scored “high”, indicating some room for improvement, particularly as resilient individuals are beneficial for safety and organisational memory in challenging environments [40]. Managers with a more senior background demonstrated higher scores for resilience – corroborating themes in qualitative data that resilience developed due to repeated challenge. There is scope for the implementation of techniques designed to develop resilience within this setting, with an opportunity to evaluate change through repeated use of the Brief Resilience Scale (BRS).

A significant number of participants felt that there was very little support provided for mangers in difficulty (a theme increasingly investigated as the interviews progressed). Managers contrasted the provision of support for themselves to that provided for clinicians in similar situations, leaving many participants feeling that they were overlooked and possibly undervalued as a professional group. A culture of not being able to ask for support was also reported, with a sense of having to “get on with it” even in the face of personal difficulty or challenge.

The findings of this study demonstrate that there is room to improve both the psychological safety and the resilience within our sample of hospital managers. It is unlikely that the concerns expressed, or challenges faced will be unique to this participant group, therefore these data are useful for all those wishing to improve the experiences of managers and their working relationships. The transferability of the data to other healthcare organisations is enhanced by the professional background of the participants. Whilst not directly enquired about during the interviews, many participants volunteered that they had worked in numerous NHS healthcare trusts and described consistent experiences across different environments and organisations. It is also likely that the perceptions described are influenced by this prior experience, and not solely reflective of the study site.

Ongoing analysis of the data set allowed the key factors described as important for organisational safety and individual wellbeing to be identified and compiled into a thematic map. This highlights modifiable areas within the workplace environment for healthcare leaders to focus upon when striving to achieve culture change, improved support and the working lives of their managerial workforce.

### Study limitations

Whilst the participant group was designed to obtain an overall and broad view of life as a manger within a large NHS trust, the lack of analysis according to specialty / department may have missed nuances that contributed to stress or psychological safety in some participants.

The study is also at risk of participation bias – those with an interest in stress / psychological safety or resilience may have been more inclined to participate, as may those who have had particularly stressful experiences within the workplace. Additionally, there is a risk of social desirability bias – participants may have presented a positive view of themselves and their behaviour within the workplace, and this must be considered when interpreting the results. The mixed data regarding the presence of both psychological safety and resilience in our participant group suggests however that the impact of social desirability bias is not a significant problem.

One research aim within this study was to investigate for differences between those managers who had a clinical background and those who didn’t. Whilst a signal that differences exist was highlighted in this analysis, the recruitment strategy was not designed specifically with this in mind, and as such the number of participants in each sub-group is not equal. This study was not powered to detect statistical significance, as such the risk of a Type 2 error is present. At best, this study demonstrates a signal that there may be some differences in the psychological safety and resilience within subgroups in our participant group. This may form the basis for future analysis in larger studies.

This study took place between February and July 2021, as the second COVID-19 surge was abating. Whilst the interview guides were designed to cover general experiences across a participant’s career and did not directly ask about implications of the pandemic, this period of increased stress and unusual circumstances may have influenced the study findings.

This study was designed as an exploratory study, and it is anticipated that further studies will be conducted in hospital managers with the aim of asking more specific research questions and building upon the findings of this study. As such, the model presented in this paper is based upon the findings of this initial exploration, and its validity requires testing in future studies and across larger sample sizes.

Given the nature of career pathways for hospital managers, the participant sample encompassed a wide experience of working in different NHS trusts. Whilst this may assist in the identification of systemic issues, we would advise caution against extrapolating across the whole NHS. The thematic framework constructed in this study could be used to evaluate other environments in a deductive manner.

## Conclusion

This study provides novel insights into the the manifestation of psychological safety and stress in managerial staff working within the healthcare environment. Notably, managers reported lower levels of psychological safety than seen in similar studies on clinical staff conducted by this research group [16]. This difference demonstrates how an organisation may have several cultures, even within the same workplace. The analysis highlights that psychological safety may be influenced by an individual’s background – managers with a clinical background scored higher for their self-perceived psychological safety.

The in-depth exploration and analysis of how managers perceive their working environment, associated stresses and the impact of this stress provide new insights into the aetiology of stress and the development of resilience. These data were summarised through the construction of a map that can be used by both hospital mangers and senior clinicians wanting to gain an enhanced understanding of the stresses faced by managers, with an aim to improving working relationships.

## Supplementary Information

Additional file 1. Topic guide for qualitative interview.

Additional file 2. Standards for Reporting Qualitative Research (SRQR) Checklist.

Additional file 3. Supporting qualitative data for thematic framework.

## Data Availability

The datasets used and analysed during the current study are available from the corresponding author upon reasonable request.
