# Supplementary material for: Exploring the working environment of Hospital Managers: a mixed methods study investigating stress, stereotypes, psychological safety and individual resilience
Source: BMC Health Serv Res. 2022 Nov 18;22:1371. doi: 10.1186/s12913-022-08812-7 (PMC9673216; doi:10.1186/s12913-022-08812-7)
Supplement: Supplementary file 1 — Topic guide for qualitative interview. [file 12913_2022_8812_MOESM1_ESM.docx]

**Supplementary File 1: Topic Guide for Qualitative Interview**

IRAS Project ID: 292036

Version 0.3 07/12/2020

**Welcome**

**Introduction & Overview**

- Introduction of researcher and brief outline of project aims
- Plan for Session
  - Interview & short written survey
  - Expected duration 30 - 45 minutes
- Explanation that the conversation will be recorded, explanation regarding data handling and anonymisation of written transcripts
- Informed consent to be taken, with review of PIS. Opportunity for participant to ask questions. (If interview taking place virtually, participant will be emailed consent form and asked to sign & email back to interviewer at the start of the interview).
- Confirm participant is happy to proceed
- **“Please feel free to ask questions at any stage”**
- **“I may take notes so that we can return to a topic later in the interview”**

**“We will now move onto the interview, which will explore your experiences working in a management role within the hospital”**

**Interview**

What does your role as a hospital manager consist of? What are you responsible for?

What do you enjoy about working in hospital management?

What is your background? (Clinical vs non-clinical)

Participants view of the clinical environment they manage:

- Explore working relationship with clinical teams
- Explore any perceived difficulties in these relationships
- Explore presence of stereotypes
  - Participants impression of medical professional priorities
  - How do they feel their physician and clinical nursing AHP colleagues view them?

Ease of implementing change

- **“Do you find it easy to identify areas where change is needed?”**
- How easy is it to initiate change?
  - If barriers found, go on to explore these
  - Explore the factors which help facilitate change
- Are you satisfied you can achieve what you intend to within this role?

Psychological Safety

**“Do you think the clinical team you manage find it easy to raise concerns / ideas?”**

- Do you think this is beneficial? Are there any negatives?
- What do you do to promote this environment?

**“Do you feel able to speak up to those more senior than you within your management team?”**

- Explore experience of dealing with senior management expectations
- Explore if they feel their concerns are acted upon
- Explore opposing challenges: financial vs patient care; organisational change vs patient care
- Explore Resilience
  - Do you find it easy to work in the face of the challenges you have mentioned?

Influence of background

- Reflect back on difficulties raised in earlier discussion
  - Does your background have an influence on this?
  - Do you think your clinical / managerial background affects your outlook and priorities within this role?
  - Do you see others with different backgrounds facing different challenges?

Closing comments

**“Is there anything I haven’t asked you about that you feel is important or a source of stress in your working environment?”**

**“Now I am going to ask you to complete a short 13 question survey, which covers two topics – resilience and psychological safety.”**

(See below for survey questions – if in person can hand paper to participant, if virtual can share screen and ask participant to complete electronically)

Once survey completed:

- Provide opportunity for any questions or further discussion
- Provide participant with the following information
  - If you wish to follow up any issues you have talked about, you can contact us (provide contact information)
  - They are free to withdraw from the study at any point and their data will not be used

Thank participant for their time.

|  | Strongly Disagree | Disagree | Neutral | Agree | Strongly Agree |
| --- | --- | --- | --- | --- | --- |
| I tend to bounce back quickly after hard times |  |  |  |  |  |
| I have a hard time making it through stressful events |  |  |  |  |  |
| It does not take me long to recover from a stressful event |  |  |  |  |  |
| It is hard for me to snap back when something bad happens |  |  |  |  |  |
| I usually come through difficult times with little trouble |  |  |  |  |  |
| I tend to take a long time to get over set-backs in my life |  |  |  |  |  |
| If you make a mistake on this team, it is often held against you |  |  |  |  |  |
| Members of this team are able to bring up problems and tough issues |  |  |  |  |  |
| People on this team sometimes reject others for being different |  |  |  |  |  |
| It is safe to take a risk on this team |  |  |  |  |  |
| It is difficult to ask other members of this team for help |  |  |  |  |  |
| No one on this team would deliberately act in a way which undermines my efforts |  |  |  |  |  |
| Working with members of this team, my unique skills and talents are valued and utilised. |  |  |  |  |  |

*Of note – the 13 questions above are a composite of two surveys. The first six questions are the Brief Resilience Scale (1), and the second seven statements are from Amy Edmondson’s Psychological Safety Scale (2). Participants were not informed as to the origin of each statement, and were presented the 13 Likert items as above.*

1. Smith BW, Dalen J, Wiggins K, Tooley E, Christopher P, Bernard J. The brief resilience scale: assessing the ability to bounce back. International journal of behavioral medicine. 2008;15(3):194-200.

2. Edmondson A. Psychological Safety and Learning Behavior in Work Teams. Administrative Science Quarterly. 1999;44(2):350-83.
