# Supplementary material for: Exploring the working environment of Hospital Managers: a mixed methods study investigating stress, stereotypes, psychological safety and individual resilience
Source: BMC Health Serv Res. 2022 Nov 18;22:1371. doi: 10.1186/s12913-022-08812-7 (PMC9673216; doi:10.1186/s12913-022-08812-7)
Supplement: Supplementary file 3 — Supporting qualitative data for thematic framework. [file 12913_2022_8812_MOESM3_ESM.docx]

Supplementary File 3: Supporting qualitative data for thematic framework

| THEME | SUBTHEME | SUPPORTING DATA |
| --- | --- | --- |
| **INFLUENCE OF BACKGROUND** | Non-clinical vs Clinical Background | “No, my background’s clinical…I do think some of my management colleagues who have come up through the management training scheme dispute this, but I think we do make, not better, but different general managers when you actually have been clinical. I think that’s invaluable.” *0095, General Manager*  “For me, it is because, basically, it’s quite interesting because I think I’ve got more empathy for them [*clinicians]*. I understand things much more. I can… But with the nurses, because I’m a nurse by background, I’m much harder on them because I know when they’re pulling the wool over my eyes, you know, about staffing levels or something. But I think you get more credibility when you’ve got a clinical background, but I think a managerial structure should, have both, people with non-clinical and clinical backgrounds, yes.” *0104, General Manager*  “I think so because I think it’s really good that they do have it. They have a better understanding of the patients, and the patient’s needs and what you are up against with your theatre lists as an example if they know what the procedures are. Yes, just even understanding the patients, particularly MDTs and that sort of thing. I think it’s good to know.” *0114, Service Coordinator*  “I think it definitely makes a difference, having that clinical background, and certainly in terms of interaction with clinical people. I guess it’s that whole thing, do you have to have had a baby to be a good midwife? I don’t know, but it really helps. Because I see my job as being able to provide the teams that I work with what they need to be able to look after patients well” *0116, General Manager*  “I find that easier, because I’ve been an intensive care nurse, and I know how to challenge a clinician about something because I’ve had to do it all my growing-up life, whereas I think some of the less experienced staff might find that quite hard. And then there are specific corporate people who just want to please their line managers and don’t push.” *0116, General Manager*  “I’m really, really fortunate that I came through the [Company] management training scheme. But you’re taught to be a leader, and leadership skills. You are not taught to be a manager. You’re not taught your really good skills in terms of any economic associated skills in terms of being able to write a business case, manage financials, have really good decision making, balance risk.” *0105, General Manager*  “In our division, there are two sorts of general manager. There are those that have been clinicians, clinical staff who’ve come up through the ranks, if you like, and then there are the… Or three maybe. And then there are the graduate trainees who are younger, and they have a completely different operating style to us gnarly old people, and then there are the people who have been in the NHS a long time but have come through the administration kind of route. There are three different types” *0116, General Manager.*  “Those that have a clinical background just have a different way of viewing things, I think. My approach is probably more structured, probably more focused on data and analytics.” *0103, Hospital Director*  “I understand their perspective and what goes on a little bit more on the shop floor maybe than perhaps some of the managers that come in who’ve just come in at that management level.” *0117, Service Coordinator*  “I see these young whippersnappers coming off the scheme and they’re fast-tracked into management, and it is all about the business and the money but forgetting sometimes what we come to work to do. And that’s to deliver healthcare and high-quality care, I think.” *0095, General Manager*  “We’ve got quite a lot of senior people and a lot of them have got clinical backgrounds. But it’s really quite interesting when you see the way things might get set up when a non-clinical person has done it as opposed to a clinical person.” *0098, General Manager*  “And the people that really stand out are the people that seem to have this amazing talent to remember stuff and be able to talk with confidence about stuff like clinical services. But I’m a bit like how do you even know what goes on in that service? Yes, and I think the people that do it well are the people that just seem to be able to assimilate that knowledge really, really quickly.” *0098, General Manager*  “I think it probably largely exists in our heads rather than in reality. So the people who come from clinical backgrounds will very often be like, well I’m a nurse and so I’m only interested in the patients and that’s my main focus. And that just really pisses people like me off, because I could have an easier life and be earning more money doing something else, but actually, that’s my focus as well.” *0100, Divisional Director*  *“*So there’s a bit of a skill set I think you learn, which is, we’re not to get involved in things. And sometimes you get that wrong. And the people who are former clinicians feel that even more because they can go and do really practical stuff, be helpful with” *0100, Divisional Director* |
|  | Significance of background on ability to perform role | “I think what’s useful is to be able to troubleshoot stuff. I think what’s useful is that I can have a conversation with someone that can get into a sort of clinical reasoning prioritisation, because I’ve been doing stuff like this for a long time. That is the useful bit, I think. And I think, generally, people treat you differently if they know that, actually, you have been there and you have seen some of it” *0094, General Manager*  “Yes, I do think so because, I can say, yes, I actually do know what it’s like, albeit 15 years ago when I was clinical, but you never forget those things. I always say I’ll never make a decision that’s detrimental to the patient. That’s not what I’m about. But sometimes I have to say we can’t afford It, but we can look at doing something this way and maybe save the money that way…..You never forget that clinical background. It’s always there, and it shapes your decision making, I think, a bit subconsciously.” *0095, General Manager*  “Yes, definitely. And especially some of the other things that we’ve taken on. For example, we often will get asked to help investigate or support a serious investigation or we’ll be involved in audit. And it’s really, really good to have the clinical background to be able to do stuff like that easily” *0098, General Manager*  *“*I think that definitely being clinical helps, really, really helps, because I think it gives you also just some of that… Just the physiology and things like that, you understand that a little bit better when people are trying to explain stuff to you. And I just think that it really helps. I think it makes you more relatable.” *0116, General Manager*  “And a manager who comes in as manager will be much more focused on maybe various outcomes but not necessarily on how to resolve things and get there in a way that works for the team or they’ll do it in a different way. But I think strengths of the NHS is the variety of managers that you’ve got.” *0107, General Manager*  “I think people who are former clinicians who go into management management, they’re not ward managers or matrons, but my kind of role, I think they do lean on it quite a lot and actually struggle to be like, actually, you’re not employed as a nurse, you’re employed as a manager and that’s the hat you need to wear. I think there are differences. I think there are things that we are uncomfortable doing. So we’re not comfortable doing that, let’s take a step back and let’s do that broader picture abstract bit.” *0100, Divisional Director*  “Because you don’t have to be clinical to be able to understand clinical pathways. However, it does help with how you communicate with people. And if you’re not familiar with the environment, there is always that oh my God, I’m talking to the consultant. Oh my God, I have to be really smart. Actually, you don’t, because the consultants, like clinicians, they’re only smart in the field that they’re in. Outside of that, they haven’t got a clue.” *0099 Operations Manager*  “And I think you need a mix, because we all need to be able to do all of those things, and you will always find people who are good at some and great at some and not so good at some others. And that’s fine, we need that sort of broad spectrum. And I actually think clinicians who go into management, management, I imagine it’s really hard for them. As a former service manager, your instinct is, right, I’m going to roll my sleeves up and I am going to go and I’m going to push some trolleys around. I’m going to pitch in and help out. You can’t always do that in these jobs now. Not because you’re not allowed, but because you might not have time or because it’s difficult for other people who are doing it. And actually, one of the things which has been difficult for people I think to admit it, various senior managers getting involved in stuff, it often makes it worse. And it’s often the doctors are perfectly capable of sorting the problem out, or they don’t need someone like me showing up where they have to spent 20 minutes explaining to me what the problem is.” *0100, Divisional Director*  “So I do think my background probably puts me in a slight advantage over possibly other managers that are grad schemers maybe” *0102, Deputy GM*  “I’ve got no business trying to leverage any clinical expertise, that’s just not my skillset at all. And those that don’t have a business background might not be so strong in business planning, but we can support each other, and peer challenge, and ask advice of each other, and it think that works pretty well.” *0103, Hospital Director*  *“*I would think there is an increasing convergence between managements and clinicians, because of being data driven. The data we’re looking at, hopefully, is well-shared. So, if you think about it, we’re trying to link both the clinical data, the access data and the financial data, so you don’t have groups looking at different sets.” *0108, Divisional Director*  “I think some of our colleagues here have a nursing background who are also GMs, and they’re great general managers, but I think the triumvirate works very well if you get one doctor, one nurse, and one manager, I think, because you all bring different perspectives towards the same goal. And I think if the triumvirate works well in cohesion, I think the success can be really, really incredible.” *0109, General Manager*  “Most managers you meet within the NHS now come through the NHS management programme and they’re fast-tracked, if you like, and they often don’t do those junior admin roles and work their way up. There are bonuses to that and I get that, but I think for me, what’s quite nice is that I can really relate to a lot of my staff because I’ve done quite a few different roles and worked in junior roles.” *0117, Service coordinator*  *“*I think what you learn on those schemes and then you transfer them to reality, it’s a very different thing. The cogs in the NHS turn quite slowly. I think they get very frustrated.” *0095, General Manager*  “I’d like to believe it allows me to have an unbiased view. It’s not formed based on my own personal experiences of working within clinical areas. The flipside of that is really I have to rely on experts and try to interpret whether the experts are feeding me their priorities or the priorities for the specialities throughout the organisation.” *0101, Hospital Director* |
| **ROLE WITHIN WORKING ENVIRONMENT** | Change – barriers and ability to effect change | “Depends what the change is. If it’s within the directorate, it’s quite easy to do” *0094 General Manager*  *“*So we changed quite a lot of things here, since I’ve been here. Certainly a lot of fabric things have been changed. Some of the problems, the reticence to change was never that people didn’t want things to be done, it was they didn’t feel they could be done. And you just have to show people that they can.” *0094, General Manager*  *“*I think most of the time if you get the right people in the room, and they don’t feel done to, that they’re part of it, it, sort of, normally works.” *0095, General Manager*  “Oh, sure. You can, yes. You can make it the priority. So that means you have the power to do that. Because what you’ll do is you’ll stop something else to either invest or resource that, to make sure that doesn’t happen. So those are the types of decisions.” *0099, Operations Manager*  *“*If it’s internal to the trust and completely within your own sphere of influence, then yes. It’s not without its technical challenges with HR and all of that, but once you understand the process, and of course you engage people both hearts and minds and their own change. Technically speaking, it isn’t that difficult to achieve, providing it doesn’t cost you a lot of money.” *0103, Programme Director*  *“*Sometimes it’s knowing who to talk to, and I’ve been here for such a long time I know most people in the trusts, so I can quickly resolve things, or they resolve them for me pretty quickly.” *0104, General Manager*  “That change has to be driven in one way, and everybody needs to be on board, so I just need to manage noise there and to manage anxieties.” *0109, General Manager*  *“*You can create your own pathway, a bit of work with it to make it better, if you see something to improve they will follow it.” *0112, Service Support Manager*  *“*it was about really getting some champions on board, and just almost circumventing the sort of professors that had been here for donkeys’ years and didn’t want to change. And that was quite tricky because people were very loyal to them. But they were working in quite antiquated ways.” *0095, General Manager*  *“*The difficulty for me is, again dipping on the external interface, is that my finances and my director of influence are completely bound up with other organisations. So, my ability to say change the discharge function. I’ve got to consult in quite a complex web with lots of other external organisations that have their own budgets, and their own drivers, and their own pressures, and their own staffing concerns, and their own HR processes, that’s extremely complicated. But it’s not impossible.” *0103, Programme Director*  “Or you’ve got a big project and you’re dependant on other people actually. Sometimes that’s frustrating because they’re not working to your timescales; they’re working to very different timescales, as such.” *0104, General Manager*  *“*Now I’m not saying that you shouldn’t have governance because you should but if you put in a layer of management to deliver something and what they actually do is talk to staff on the ground and actually waste their time. So they talk to the staff about something that the staff know and they’re forever talking to staff and forever talking to staff and then you’ve got somebody else that does the same thing, it just elongates every process that you’re doing and people get tired of that.” *0107, General Manager* |
|  | “Helicopter” view of working environment | “The clinical direction I do more than strategic stuff. So, we’re doing the really big, sort of, stuff where we’re constantly having this helicopter view” *0095, General Manager*  *“*And I think that’s, in some ways, you can’t criticise for them for that because they’re only seeing the bit they see… And our job is to have that overarching responsibility and accountability, but sometimes it’s a bit difficult to explain that maybe you’re not the priority” *0095, General Manager*  *“*And ultimately, I came in on that and just like, this is what I see as the overall piece. So, if this is the overall piece, which way do we go to get to an outcome and an answer without directing people as to what to do. But just say, look, I understand what you’re saying, but I’m not just thinking about this bit that goes from here to here to here, I’m thinking about this bit, so I want this to go from here to there” *0096, General Manager*  *“*I think my job is to make sure people are aware of those [unclear], because that’s their role. So my job is to make sure that level of communication does pass through, so there’s awareness and empathy. To empathise in a way that this doesn’t stop here. So if I’m going to do something now, it could impact somebody else down the line. Just that understanding the whole journey” *0099, Operations Manager*  *“*So, I think my role, ideally is to bridge those divisions in the speciality discussions to make sure that what is optimal to one service is actually not having a detrimental impact on another and vice versa” *0101, Hospital Director*  *“*I think that’s often when I see conflict is. I’d see it so often and so clearly, of someone comes from it from one side, and they see their bit of the world, and they see it as really simple. And someone’s from the other side, and see their bit of the world, and it’s so complex, so how could you do it? That’s pick up the feather and just lay it all out. You want to solve the problem relatively easily” *0105, General Manager*  *“*Yes, I think you have to remind people of the things they need to know, like that. They might say, why don’t we do it this way? And I can say, well, the thing you need to know is, this. Why can’t? Well, there’s actual money and there’s revenue money, and it’s trying to explain to people whey they can’t just spend capital money.” *0106, General Manager*  *“*it’s that overview of the whole service” *0096, General Manager*  *“*So, basically recognising that each of our three, four sites have got different cultures, different priorities, different focus areas. And recognition that it needs a leadership team that have got responsibility for knitting and gelling all of the specialities that are on a specific site.” *0101, Hospital Director*  *“*So, I think my role, ideally is to bridge those divisions in the speciality discussions to make sure that what is optimal to one service is actually not having a detrimental impact on another and vice versa. And ideally, we’ve got a win-win with decisions that are being made at a local level.” *0101, Hospital Director* |
|  | Role as facilitator | *“*Actually, a lot of the first intro is trying to convince clinicians, nurses, AHPs, whoever, that actually, I do care as much about patient care as they do. I’ve just got a different role which is more of a facilitative role rather than an actual caregiving role.” *0113, General Manager*  *“*So, divisional or wider organisational meetings where you’re representing your whole service, whether that’s nursing, medical, whatever function you’re representing, that articulating the passion, or the energy, or the frustration that they have into an appropriate way to share that message, can be quite difficult” *0096, General Manager*  *“*I suppose that it is a bit of managing unrealistic expectations about how quickly things can be done or actually what we’re trying to improve doesn’t actually sit with us. We’re an enabler but we don’t make it happen.” *0098, General Manager*  *“*, I think my role, ideally is to bridge those divisions in the speciality discussions to make sure that what is optimal to one service is actually not having a detrimental impact on another and vice versa” *0101, Hospital Director*  *“*Well, they don’t know how to access the organisation. I think that’s what I’m improving. I understand who you’ve got to go to and how you will block things. I very much see it as my role, as helping to unblock things for people so that they can go and do what they want to do.” *0105 General Manager*  *“*Doctors are obviously really, really clever people, they know their services really, really well, and I see myself as more of a facilitator sometimes in terms of trying to do what they want me to do.” *0117, Service Coordinator*  *“*I see my role as being able to facilitate some resourcing that they need to be able to do their job, that’s a very big part of my role” *0116, General Manager*  *“*I think, also, there’s a view that it’s my job to do it for everybody. Whereas, actually, for me it’s about empowerment and self-education. I’ll say, okay, well, I’m not going to do this because, actually, we need to get clinic space. Or, you’ve another manager I can ask, can help you.” *0105, General Manager*  *“*You do have a lot more exposure, because there is a lot of… Everyone’s looking at you to be able to help them deliver their service. We’re in quite a unique position in terms of how we network with other people and try to help them in the form that they need to deliver on their targets effectively.” *0110, Business Manager*  *“*And I also found that clinicians, when they all complained a lot, if I sit and I give them all of these explanations, they will go away and find a solution themselves. They don’t necessarily want me to resolve the thing for them. They just want that permission” *0105, General Manager*  “So my job is to make sure that level of communication does pass through, so there’s awareness and empathy.” *0099, Operations Manager*  *“*I think there’s a lot of me, the peacekeeper, and keep people calm, and do that.” *0102, Deputy General Manager*  *“*I think, upfront I usually say, look, my deadline is X, and probably yours is Y, but if we can try and meet in the middle and understand where I’m coming from, so explain to them why because I wouldn’t just have a deadline for a deadline’s sake” *0104, General Manager*  *“*I’d see it so often and so clearly, of someone comes from it from one side, and they see their bit of the world, and they see it as really simple. And someone’s from the other side, and see their bit of the world, and it’s so complex, so how could you do it? That’s pick up the feather and just lay it all out. You want to solve the problem relatively easily.” *0105, General Manager* |
| **THE PRESENCE OF STEREOTYPING** | Presence of stereotyping and persistent stereotypes | “But, yes, it does my head in when they say the managers, and they’re like, right, yes. It’s a massive term to use, isn’t it, in the NHS because we’ve loads and loads of different managers.” *0095, General Manager*  “So, clinicians will think, oh, this is about, to make them all balancing the books. Whereas, actually, it’s a real opportunity to set out what you want. Any business plan is about what you want to do. What you want to do on behalf of your patients and your people, and what that means to your bottom line, in terms of money.” *0108, Divisional Director*  “They also look at you a bit in terms of your age as well. I think if you’re seen to be a young manager, they automatically assume you’re not going to really be around for very long. I think that also is a bit stereotypical. And I think there’s always been… I do feel that sometimes there’s… There’s always been a bit of a strained relationship, I think, with consultants and managers in the organisation.” *0110, Business Manager*  *“*I think the doctors know that I’m like that, now, and I won’t be pushing things just for the sake of it or just to hit a target, that I’m a bit more pragmatic in that approach. But I think anybody coming in new, yes, I think they are very cynical and just think, oh, they’re only interested in hitting their goals, and we’re interested in our goals, and never the twain shall meet.” *0111, Business Manager*  “The NHS is charged with making cost savings every year, it’s a political thing. It’s not a thing that managers want to do but it’s viewed as something hospital managers want to do.” *0113, General Manager*  “I think that there’s definitely something in that. It’s not completely unfounded” *0098, General Manager*  “Most people, when I did it, were, like, oh, you’re going to the dark side of management.” *0095, General Manager*  “But there was definitely an element that they’d already decided that there was a bit of corporate services that don’t really do any work compared to the people that are really doing a lot of work on the ground.” *0098, General Manager*  “I do think we are perceived in that stereotypical way, yes.” *0110, Business Manager*  “Yes, I guess so. I am not too sure. I think yes, you just have to be very focused. It seems very target-driven. I think there is that stereotype there. I would have to agree with that.” *0114,*  *“*Do I come up across that stereotype? Yes, I do from the clinical point of view sometimes. It’s harder for them to see beyond what’s just happened in their clinic, what happened when they were attending last week, and can’t see a bigger-picture view of how we might change something to make them feel better about how they’re practising.” *0116, General Manager*  *“*Quite a lot of cynicism in some teams about, well, let’s try this, oh, trying something, we know it’s not going to work, da-da-da, all that the trust cares about is money, it doesn’t care about patients, says it cares about patients but doesn’t really care about them. I do see some of that, but then, saying that, there are other clinical staff who I’m surprised at how interested they are about what goes on with the finances, what that means.” *0116, General Manager*  “It feels to me though, that as my career’s gone on, I come across the stereotype less and less” *0100, Divisional Director*  *“*My personal view is it’s slightly outdated.” *0101, Hospital Director*  “I think it’s really outdated. Because actually if people know my role, I know all the risks on our risk registers” *0102, Deputy GM*  “I think it, in the main, it is outdated now” *0103, Programme Director*  *“*I think it’s more outdated.” *0104, General Manager*  *“*There are a few traditional managers around who will say, well, basically, you know, if a clinician said I need X, Y and Z, they’d say, no, no money, whereas a lot of us are very much about let’s work this through, let’s see how it can, even if it’s an averse [unclear] see how we can try and make it work. So, I think gone are the days where there was the traditional, kind of, finance pay-at-the-month-end managers rather than just clinicians as well.” *0104, General Manager*  “, I think they’re, well, I would hope they’re outdated.” *0108, Divisional Director*  *“*So, my view is, it is outdated, but perhaps not as outdated as I would hope. My experience has been, and what I have fostered, hopefully, has made it outdated.” *0108, Divisional Director*  “I hope it’s outdated, and it should be outdated, but I know lots of my colleagues who are managing exactly like that, general managers who won’t get involved in anything that doesn’t have numbers next to it” *0109, General Manager*  “So, we worked quite closely alongside each other since the day I got here, and one of the first things he said to me was, management never stay. This is a consultant-led kind of environment because management just don’t stick around. If I don’t get on with management, I’ll wait until they disappear, and the next lot to come in in 18 months’ time and we’ll just carry on that way.” *0096, General Manager*  *“*I feel that also there is a bit of a stereotype that managers don’t stay around for too long as well. I remember when I first joined, and I think there were a couple of times where something the director actually said to me, that, oh, have you got a plan for handing stuff over” *0110, Divisional Director* |
|  | Reasons for stereotyping | “And one of the things that all of the trainees used to say when we went through, was that they didn’t actually understand that managers really were there for the patients. They thought that they were purely there for money. And that’s not the case.” *0094, General Manager*  “I know that there are general managers who do not get involved in anything that is not numbers, and everything that’s clinical or safety-wise, they will just push back.” *0109, General Manager*  “But people only see the hospital manager group rather than the overarching groups. Then if you look at management costs as a whole it feels the public perception is well, there must be so many managers in hospitals.” *0113, General Manager*  *“*I think the reality is, and a massive issue, something that I would like to go on to do in a later career, is actually, it starts with education. And, if you look at the medical training, you’re taught to be a doctor….Yes, so how can you then turn around and say, well, they never get involved, when you’ve not even been aware of this whole other world that exists.” *0105, General Manager*  “But if you are a doctor or you’re a consultant and you have a really bad experience with a manager, you might only come across a couple more managers in your life. It’s not inconceivable in some organisations, that every single one you come across doesn’t care about patients, because in lots of ways, lots of doctors don’t care about patients really. Lots of managers don’t really care. I think a lot of them have had this experience, they’ve passed that experience onto their teams and then breaking that down can be quite challenging.” *0100, Divisional Director*  *“*People will introduce you as, oh, you need to speak nicely to [Name] because she’s the one with the money. And I think, the other thing, the one thing, I’m pretty even tempered. I’m experienced and I’m even tempered. I don’t tend to get excited about stuff. The one thing that will always rile me, is when people tell me how they care about the patients, as if I don’t.” *0106, General Manager*  “So, I think, yes, that we do come across it a lot. And, you almost have to make more of a deal of it, because I think people expect you just to be interested in money. But of course, if you look after the money, we can provide care for more patients. So, yes, actually I’m worried about all the patients.” *0106, General Manager*  “one of the reasons is that they’re patient focussed, whereas we’re money focussed or finance for me driven, focussed.” *0110, Business Manager*  “I think it’s this sort of generic term which drives me mad because I’m saying if I haven’t done something, tell me it’s me, the general manager. But often it’s like when you drill into it, it might be, like, the bureaucracy that’s stopped them doing something. It’s not always me, but it is, some of the processes are bureaucratic, and that frustrates them.” *0095, General Manager*  *“*But I think that’s people not understanding we’re responsible for the governance, we’re responsible for the quality and safety. It’s a lack of understanding.” *0102, Deputy General Manager*    “Yes, a lack of understanding and a lack of education.” *0105, General Manager*  “And that’s the issue that some have where their manager’s always looking at it at a macro scale and the individual clinician over 20 years perhaps of fellowships and research years and core training is all focused on how do you actually do the surgery? How do you do the…? And that’s real imbalance and it’s only later on down the line that it appears that goals are not aligned. The goals are aligned it’s just two different lenses that you have to look through.” *0113, General Manager*  “I think everyone thinks that what I do is really easy. Some of the clinical staff that I work with, they think, oh, what does she do all day? That just shows a lack of understanding, and sometimes when you’re challenging back about some of this stuff, I think they find it really quite hard.” *0116, General Manager.*  “Then your clinicians worry that managers come and go. They’re there for two years. It’s one of the big concerns. If you listen, speaking to them, they’re there at the start of the business planning cycle. They’re never there at the end. They’re never at the start and the end of a business case.” *0108, Divisional Director* |
|  | Influence of individuals priorities – financial vs clinical | “On the clinical side, I think it really varies. I think there are some clinical directors and [unclear] in clinical leads departments [unclear] who are very switched on to the business side of things. And because they’re genuinely interested in it, or they’re confident talking about that kind of thing. I can see some who have risen to great heights who have no interest in that area and their general managers compensate for them.” *0103, Programme Director*  Some of them [*clinicians]* are quite selfish and throw their toys out of the pram, asking for something for themselves rather than the team. But you can read them a mile away.” *0104, General Manager*  *“*Whereas, not that you’re not, as clinicians. But you’re worried about the patient in front of you at the time. Or, your group of patients. Whereas, I have to worry about a big group of patients, and making sure that our consultants have what they need, and all the rest of it, to look after them.” *0106, General Manager*  “I think the consultants are more interested in the patient, absolutely and they don’t want to learn about finance but they recognise that finance is there but they expect somebody else to deal with that.” *0107, General Manager*  “But overall, I think financially, everyone knows we are financially driven. Our decisions are often led by the financial ability to what we have. And also, it’s also balances on the types and numbers and complexities of patients.” *0099, Operations Manager* |
| **WORKING RELATIONSHIPS** | Building and maintaining working relationships | “Potentially, that’s something that maybe is drilled into us from a graduate scheme, because you have to have, you’ve got a mandatory one-month orientation period at the beginning of your scheme. So, I applied to spend time to clinically. I would work in quarters. I was in the kitchens. You really get to know the bowels of your organisation and how it’s run.” *0105, General Manager*  *“*I think if you come into it just looking purely at the finances and don’t do that, then you don’t then get… You really do need those working relationships. And that’s something that I’m always really keen to do.” *0117, Service Coordinator*  “So, I’m really open and honest with my clinicians, and we share, I share everything. I say there are no secrets, and the budget is the budget. If we haven’t got the money, we need to work out how we get it. We write business cases together. So, I’m really transparent because I think it’s a much better working relationship.” *0095, General Manager*  “I used to as a much more junior manager, but not so much as I’ve got more experienced because I think you have to very much work together. It doesn’t really work if I’m always talking about the money and they’re always talking about quality care. You have to really work together, and, you know, you have to educate the clinicians about the sort of budgets and that there isn’t a bottomless pit of money.” *0095, General Manager*  *“*Managers come and go, but the clinicians tend to be there for a long time, and it’s their service and they’re the ones that are usually really passionate about it. And you really have to build your relationships with them in order to really drive through the changes and the improvements that you want.” *0117, Service Coordinator*  “So, I would never go out and say no, I’d always have a caveat behind that, or sit them down and say what’s your rationale. So, we work together to try and achieve it. Yes.” *0104, General Manager*  *“*So, you have to work alongside each other. We definitely have times where we don’t always agree. But we work through them” *0096, General Manager*  *“*I think that there is a recognition that from a whole system perspective, certainly that we have here, that there’s a mutual respect and admiration, I think, from the management, and the clinical, and the nursing side, about how we’ve all got our own way of being involved to run the directorate as a whole.” *0096, General Manager*  *“*I have really, really good relationships with the clinical teams. I think they’re only key people on. You don’t really see everyone. But I’ll have contacts with the clinical director every day.”*0102, Deputy General Manager*  “Because I’ve always had really great relationships with my clinicians. I’m not sure why that would be the case, but I think it’s really key. My portfolio is everything. I think, for me, the first rule of business is, know your business…..And, I think that’s really key. And just that then, trying to find common ground and shared understanding, is essential in terms of building relationships, but also being able to manage any portfolio that you’re responsible for.” *0105, General Manager*  *“*Something that I’ve learnt, I suppose, from being a manager is to really, really work with your clinical team and get to know them first.” *0117, Service Coordinator*  *“*So, everyone has their own priority and when you’re a manager who is a support service manager you’ve got every other area in the Trust and outside of the Trust who think their individual patient is the highest priority there is ever. So, managing expectations and managing the whole communication chain is quite important.” *0107, General Manager*  *“*I’m really open and honest with my clinicians, and we share, I share everything. I say there are no secrets, and the budget is the budget. If we haven’t got the money, we need to work out how we get it. We write business cases together. So, I’m really transparent because I think it’s a much better working relationship.” *0095, General Manager*  *“*I’ve never, ever said to my clinical director, absolutely not, we’re not doing that. I listen to him, and he’ll guide us all the time about the right clinical decisions. I always say we’re not in the business of making car parts, and it is healthcare.” *0095, General Manager*  “I think visible presence is important, probably you know what you’re talking about is more important. But, or if you don’t know what you’re talking about, admit it and take advice from someone that does. But yes, I do think it’s important.” *0094, General Manager* |
|  | Presence of conflict | “I think there is always conflict, but I think it depends on the culture that is around you or you’ve developed in the service that you’re managing that says, do we let finance define what we do? Or do we let patients safety drive the developments and everything that we need to change or do or deliver in the service? But the financial is a big conflict area that we have to manage. The other area that I would say takes quite a lot of energy is managing expectations and setting expectations.” *0107, General Manager*  *“*it’s going to be in relation to somewhat patient care or whether it’s… One example is reverting from face-to-face interpreter to a [unclear] interpreter and see what savings are there, but then the quality of interpreting goes down. It’s those kinds of little things that we would as managers be looking at, but someone clinical would say, actually not really in agreement with that. But those are the kinds of challenges that would be face to face” *0109, General Manager* .  “So, I want to improve things for patients by using this. Well, there is no money to buy it, so you can’t. Well, it doesn’t matter. You can, you know. There’s nothing you can do.  So, yes, there is a conflict. Definitely. Definitely. But you could give the banks a try. You can get the banks to try at customer care. Kindness. All that. All that stuff. All that rubbish.” *0105, General Manager*  *“*I think there is always conflict, but I think it depends on the culture that is around you or you’ve developed in the service that you’re managing that says, do we let finance define what we do? Or do we let patients safety drive the developments and everything that we need to change or do or deliver in the service? But the financial is a big conflict area that we have to manage. The other area that I would say takes quite a lot of energy is managing expectations and setting expectations.” *0107, General Manager*  “So if you want a job, you have to write a paper. It then goes to a divisional group, and then it goes to executive group. And then it goes to someone else. And this doctor didn’t get why the process couldn’t just be tomorrow.” *0102, Deputy General Manager*  “Whatever they have to throw at us, bad behaviours, capital letters, highlights, my job in their eyes is just to take that because they were upset at that moment, and I think they forgot that we’re also people and that we have our own problems in our own lives” *0109, Divisional Director*  “but it’s just making sure you’ve got those good relationships up the line, I think, as well. You get the odd doctor writing to the chief exec, and then that annoys the chief exec. And then he writes back to you, saying you need to manage your doctors. And you’re like, oh, God, it’s not for the want of trying.” *0095, General Manager*  “The difficulty is obviously you get different opinions between doctors sometimes. That’s quite a difficult line to manage sometimes, but it is really, really key, those working relationships,” *0117, Service Coordinator*  *“*as a junior manager, there’s very little respect, certainly from senior clinicians, as to what the role is or what value that it adds. It’s a means to an end.” *0096, General Manager*  *“*If the clinicians wanted it changed, they expected it to be changed, regardless of what the principles were about any of it. And they were just talked down to to do what was asked, because we’re the clinicians and you do what we asked” *0096, General Manager*  *“*And that frustration gets vented towards operational managers……That never goes well, because then, it becomes oh, you don’t care about us, you promised us this. And oh my God, you’ve failed us.” *0099, Operations Manager*  *“*Sometimes you get consultants or nurses who, sometimes due to an experience or sometimes due to frustration, feel that it’s the wrong decision. And clearly trying to manage that reaction is sometimes tricky. But I think to a certain extent it goes with the turf, unfortunately.” *0101, Hospital Director*  *“*Consultants think that they have a right to write an email with capital letters, underlining letters. I get an email with capitals, underlined, and then highlighted…” *0109, General Manager*  “But that I think it’s a bit of a challenge where you’re having to deal with a hugely intelligent group who won’t respond well to authoritative leadership styles. Because they go, well I’m internationally known for this and who do you think you are? Whereas if you can have a more influential and at times even coaching when it comes to management aspects, you can either help them see your point of view or give a bit of a softer blow. But these people are your… I view them as colleagues, but there are some managers who don’t, but I do.” *0113, General Manager*  “But on the odd occasion, I have to step up and say, right, I’m really sorry, guys, this is the decision we’re going to make, but I find most of the time you can come to the decision together.” *0095, General Manager* |
|  | Working in a triumvirate | “Because I think it’s good to have three different views, and sometimes we go too much with patients, patients, patients, and it’s absolutely in the centre of everything we do, but it’s also being reasonable with it and what is reasonable and not.” *0109, General Manager*  “I’ve always worked as part of a triumvirate in a division. So, there’s always been me, a divisional director who’s a manager, and then a clinical director who’s a doctor. So, we’ve always worked quite closely together.” *0098, General Manager*  “it’s normally been built around the triumvirate, as [Company] has. Because normally there’s a lead clinician, consultant, doctor, and there’s normally a lead nurse. And those three together I think how they interact and how they work together forms the baseline around how the whole directive works.” *0101, Hospital Director*  *“*I don’t know if I’ve just been fortunate. My experience has been those three have always worked quite effectively.” *0101, Hospital Director*  *“*I don’t know if I’ve just been fortunate. My experience has been those three have always worked quite effectively.” *0103, Programme Director*  “We had to do a very big piece of work together to find where the best strengths of collaboration was. And, I would say every triumvirate has to do the same.” *0108, Divisional Director*  “The interesting one, back in it, we don’t recruit triumvirates as in, trying to consider what that team skill-base is, and it’s something, I think, we should do more. We should really consider, in terms of, what is the fit? Our recruitment strategy shouldn’t be just based on the job description. It should be based on what that team-based skill base is, and what is it we need for that next two to three years” *0108, Divisional Director*  *“*I think the triumvirate works very well if you get one doctor, one nurse, and one manager, I think, because you all bring different perspectives towards the same goal. And I think if the triumvirate works well in cohesion, I think the success can be really, really incredible.” *0109, General Manager* |
| **PSYCHOLOGICAL SAFETY** | Perception of own Psychological Safety | “Yes, definitely. I can raise them to our divisional leads. And by the time I’ve got a problem, I imagine it won’t be unique. Because if I’m facing it, it will be something that’s already filtering through. So the first thing would be to check that somebody else had this problem. Oh, maybe I could have learned before escalating. So there’s something you can do for yourself first, is to check. Because at the end of the day, it doesn’t matter which service you’re in. As a general manager, you will be dealing with similar things in different ways as your counterparts and colleagues as well.  So there’s definitely a shared learning there. But yes, I can raise concerns, issues, just a white flag if it’s getting a bit too much at any time.” *0099, Operations Manager*  “And you feel much more comfortable owning that you don’t know. I think you learn a little bit about your audience and who you would go to with what.” *0100, Divisional Director*  “But I would talk to my immediate boss about it who’s also an executive, and I would feel comfortable putting my hand up in a big executive meeting saying, we need to remember this and what do we think about that. You just sort of get better at being your own internal filter a little bit, about what you do and don’t bring.” *0100, Divisional Director*  “And both of those individuals I’ve got full confidence in, which does make my position healthy and strong I think in terms of whether I feel comfortable speaking out.” *0101, Hospital Director*  “I feel comfortable with raising them. I don’t know if they’d be acted upon.” *0102, Deputy General Manager*  “I did have a serious concern that needed executive input, I would go via our divisional representatives, who are great.” *0103, Programme Director*  “I think it depends which executive you’re talking to. If I wanted to raise a concern, yes. if I wanted something done about said concern, that responsibility firmly sits at my feet.” *0105, General Manager*  “I’ve learned that there is, probably, over time, there’s probably ways to do it. So, I think you have to, when you’re in these big organisations. What am I saying about this? What do I think?  I think you have to make a determined effort, sometimes, actually. It’s not an easy thing to do. In that context, if you can make a determined effort, then it doesn’t feel like the safest thing to do, is my view.” *0108, Divisional Director*  “No would be the answer, but that’s not because I think I’m going to be fired or I’m going to… Nobody’s going to do anything to me, if that makes sense, if I speak up. It’s not one of those things like somebody’s going to fire me.” *0109, General Manager*  “I think within our own team, yes, I would be comfortable raising it within our own team, but I think maybe more at a senior level, probably not, because I think within our team, it goes as far as our team, but then I don’t think maybe further than that.” *0110, Business Manager*  “So, on the whole, it would be with my direct line manager, but I have been involved, occasionally, with the execs. I wouldn’t feel quite so comfortable in raising difficult issues there” *0111, Business Manager*  “No, I don’t. It’s a bit tricky. I think it depends on the person” *0114, Service Coordinator*  “Yes, I would do. I think it’s really important that everybody, no matter what their role, feels that they can have a say and can have an input and make changes. Yes, it’s something I would be really keen to do.” *0117, Service Coordinator*  “I suppose I’ve probably got confidence from having been in the NHS for quite a number of years to speak up, and I certainly wouldn’t worry about voicing concerns. I think, yes, I would feel comfortable about having those conversations, and if I had things that worried me, then I wouldn’t feel that I couldn’t approach senior managers about it” *0117, Service Coordinator*  “No, I think I’m going to continue to do it. Because the way I think it’s right to continue to raise it. But I’m realistic around there’s probably nothing that’s going to happen from me doing this.” *0102, Deputy General Manager*  “Yes and no. I know that even if I will speak up nothing will be done. So, there’s no point of doing it.” *0112, Service Support Manager*  “But I think there’s a deep-rooted feeling, for me, is that I will never escalate anything. And that could be me. It’s not because I can’t. It’s me.” *0099, Operations Manager*  “And I’m trying to make suggestions to my line managers, and they don’t want to hear those because that’s too hard” *0116, General Manager* |
|  | Barriers to Psychological Safety | “I won’t escalate anything unless I completely try to fix as much as I can of it first. Because you never want to be the one who just passes on the problem” *0099, Operations Manager*  “I think it’s quite tricky outside of that. I would worry about things people say politically. I think there’s been a couple of incidents where someone said something innocently. It was meant well. It was a good idea. But depending on who you are around, and who it was said to outside of [department] it’s come back to bite us a few times.” *0102, Deputy General Manager*  “It’s difficult, you can’t just go and drop by people’s office anymore and the big group meetings, obviously, are no longer possible in the same sense. And those informal interactions where you can voice a concern are impossible to achieve really anymore”, *0103, Programme Director*  *“*Rather than asking me is there a problem, what are you doing about it, how are you managing it, I was told, there is a problem and it’s your fault. Now there is no psychological safety in that environment” *0107, General Manager*  “So, it didn’t feel you could hear everybody’s voice in there. And actually, it didn’t feel psychologically safe. The fact that you have 40 people in. There’s no way. You don’t know them all, actually. So, I feel it is the numbers.” *0107. General Manager* |
|  | Organisational Culture and Hierarchy | “No, not really. And I think that’s quite common I’d say at the organisation that people don’t feel comfortable about that.” *0098, General Manager*  “With my own divisional structure again within the last couple of years, the divisional team have definitely tried their hardest to create a place where people can bring their concerns and be themselves.” *0103, Programme Director*  “In this trust, it’s much more difficult.” *0116, General Manager*  “I think there is still a bullying culture in my environment from the team above me and I see it or hear about it a lot. That’s not good either.” *0107, General Manager*  “I don’t think that there’s a strong culture of standing up and saying this isn’t right or I don’t agree with it or is there a better way of doing it.” *0098, General Manager*  “I don’t think the culture’s there at all. And I think that you definitely see a lot of bad behaviour. And, yes, we’ve done so much work around culture and behaviours and values, but it just seems ingrained in some people that they just fake it for whatever purpose that they need to. But actually, deep down they’re not going to change.” *0098, General Manager*  “A lot better than it used to be. I’d say there’s been a kind of cultural shift in the last five to ten years. It’s still not perfect, but it depends on who you talk to how receptive they are. But, generally, I think there’s much more willingness to listen at least. Yes. there’s been a sea change, yes.” *0104, General Manager*  *“*I think it’s just a real, acute trust culture, where you think, if you speak out, that’ll be it. You’ll be, you know. Just get on with it” *0106, General Manager*  “And there is still a lot of… Well, it’s not finger pointing but this is your service, you should be managing this. Well, actually, they had created the problem but hadn’t recognised that this problem that I need to be aware of, don’t leave it until you’ve got a second follow-up meeting or a third follow-up meeting with an individual that says I’m not sorting it out. Tell me that there’s a problem that I need to be aware of that I can actually do something about. Don’t assume it’s my fault 100%.” *0107, General Manager*  “I will say something, although I don’t think that that culture is welcomed” *0116 General Manager* |
|  | Fostering Psychological Safety in teams | “If they need a hand they can ring me anytime they want. They very rarely do, but quite often they will ring and they will give me what their solution is, because they just feel that they need some support. And of course, the more support you give, the less they ask, because they know that they’ll get it, and that’s important.” *0094, General Manager*  “So, I really try to lead by example. But also, I try to be really clear that people can come to me, that this is what it’s done for. But also, to walk the walk as well and make sure that I am doing it and not just saying it.” *0098, General Manager*  “Always. Always. I think it’s really important to know the whole of your team end to end where you can. The open door policy, always had an open door policy. It’s like Piccadilly Circus, but nobody should be afraid. It doesn’t matter what grade band, whatever you are, nobody should be, or is, afraid to walk in” *0099, Operations Manager*  “Yes, in so far as I’m very clear with them and if we have a conversation, it’s around, you can come to me with anything. I would never turn around to somebody and be like, I’m not interested in what you’re telling me, go away. Sometimes conversations go down the road of, you need to fix this and I’m going to help you figure out how to fix it. It’s rare that it’s like, I’m going to fix this for you, because you’re normally talking to quite senior people” *0100, Divisional Director*  “I’m conscious that the leadership responsibly is to be open and have people able to speak about anything to me or with me. I’d like to think that I do create that opportunity and that culture within the site, and I have done that over the last 12 months.” *0101, Hospital Director*  “I did it in a way that was exactly designed to create psychological safety. And for people to really share their fears, and their anxieties, and their aspirations, and their all sorts of feelings about what this future might hold. And about the process that they were going through at the same time.” *0103, Programme Director*  “I would like to think my team think there’s a culture of psychological safety. But it’s about saying, let me know if there’s a problem. Just come in. And, just checking in with people and saying, is everything okay?” *0106, General Manager*  “I personally do and particularly encourage my team, the team a level below me and a level below that to do the same, to get the feedback and get, how can we change things, how can we do things? It’s not always easy.” *0107, General Manager*  “I do believe in a completely flat structure. I want to believe that I nurture a culture where everyone can speak up. From my experience, I can tell you that my managers are speaking up a lot.” *0109, General Manager*  “But I also have an open-door policy. Whoever is angry or upset at work, they just come in, and I listen and I take it for them and then we move on.” *0109, General Manager*  “I think I do try to promote an open culture, but it is such a large team that I don’t know if everyone would feel the same way, if I’m honest.” *0110, Business Manager*  “I don’t have the answers to everything at all, and I think if I can’t create an atmosphere by which my teams can challenge me, then that puts patient safety at risk.” *0116, General Manager*  “Yes, I definitely feel that I try my best to ensure that that’s a culture we have as a service so people can say, I don’t think that’s a good idea, or have you thought about this, or have you thought about that? Definitely.” *0116, General Manager*  “And I would always encourage my staff to speak up, and if they want to be part of meetings, I would always definitely encourage that. *0117, Service Coordinator* |
|  | Experiences of negative consequences of psychological safety | “Yes, all the time. All the time. It doesn’t last for long, but it’s almost like I’ve opened the door to you. The least you can do is check I’m not on the phone or something. But it’s fine. They know. And it can be, because then, you sometimes don’t have enough time to do everything else” *0099 Operations Manager*  “Yes, it has quite a few downsides. There’s a purely personal one, which that it can be really exhausting. So if you set yourself as a, I’m going to be someone who anyone can speak to, you have to be willing to accept the fact that anyone might come and speak to you. As well as taking up a lot of time, which is fine, you can manage that, it can be really difficult to absorb all those other issues.” *0100, Divisional Director*  “From a member of staff’s point of view, you can also, by accident as well, have a bit of a dependent relationship. Then when you try and cut that off, you’re like, you don’t need to talk to me about this, just go and do it, it can be quite difficult. So there’s lots of downsides, but it’s quite difficult to predict, it’s all about personalities and circumstance” *0100, Divisional Director*  “Yes. In terms of resilience, it can be quite difficult. And it’s difficult to separate out what someone’s moaning and griping, and you just need to compartmentalise and let them offload. And then, versus something that you’d have to do, figure out. Because if someone, understandably, is complaining consistently about something, you feel really accountable and responsible.” *0105, General Manager*  “There are difficulties because I’ve got in my service quite a significant number of consultants and if you have three consultants in a room and you ask one question you will have four different opinions.” *0107, General Manager*  “It’s about trying to find that little balance], and it is quite difficult to find that balance”, *0110, Business Manager*  “I know, and I don’t have time just to sit and have a chat about your daughter, or if I do, I’m hurrying you along. And I really don’t like that, and I will say to people, oh, but I don’t want you to feel that about me. You can come and talk to me.” *0111, Business Manager*  “Yes and no. A lot of questions is fine, actually. And if you can tell people why things happen the way they do and why you have to be focused on the things you’re focused on, it may help them understand where do they fit into your life? I get loads and loads of aimless complaints of just general moaning. It’s often hard to differentiate is this an individual coming to me who just wants to moan, where you can sit and listen to it and go, I know, there’s crap all over.  Or are there unreasonable pressures put on management colleagues to fix things which are inherently flawed and cannot be fixed? That’s the two differences. If they’re wanting to come and have a chat and complain all about that I have three chairs in my office, two chairs are positioned where if you walk past my office you can’t see the person in the chair. So, some people just turn up and have a moan. I just say, you can sit in the chair.” *0113, General Manager* |
| **RESILIENCE** | Presence of resilience and maintaining resilience | “I’d say, probably quite a resilient person, quite a mentally resilient person.”, *0096 General Manager*  “And you teach your team how to be resilient about it. We can’t fix everything. That’s what this is about. You’re not going to get it right all the time. You are, sadly, going to get something wrong or mess something up. Or it didn’t go in the plan you wanted it to go in. But you learn from that. And for me, a failure of that would be something like failing a timeline or failing at patient care. Not detrimentally or fatally but could be detrimental…… And this is where your bigger picture comes in. Because the resilience is yes, yes, yes. You’re going to give me that, but actually, I’m going to make such a difference here that when I leave today, I’ll have no mental breakdown that I’ve failed something. Because I’ll deal with you tomorrow” *0099, Operations Manager.*  “So I think there’s a bit of me that goes, what does personal resilience mean, and what I realised is that I guess number one, we often as managers or as leaders mistake the ability to get through the day as being resilient. You can show up to work, you can get through all your meetings, you can be good at your job, but that doesn’t actually mean you’re being resilient, you might still be holding quite a lot of anxiety or whatever. But the other bit which is probably a bit bigger, for me anyway it depends almost entirely on where I’m working and who I’m working with and the cultural level the organisation has got. And that makes you more resilient because you’re walking round carrying less anxiety. So I think a lot of it depends on your organisational culture, which I think is really hard to measure.” *0100, Divisional Director*  “I think, that’s something that I’ve learnt is, if someone gives you a bit of feedback, that’s their opinion and it’s one day of their life and your life, and it’s not necessarily the truth. There might be truths in it, but you’ve got to decide what you do with that information, you don’t have to internalise it. So, that’s a bit of resilience, I guess I’ve improved this last year, yes.” *0103, Programme Director*  “I will always take difficult challenges. But there also bits where I’ve been more mindful, actually, that I don’t take on stuff blindly, without even thinking about the consequences of going to be onto myself.” *0108, Divisional Director*  “Yes, I think I probably have become more resilient. I think I’ve learnt to seek support as needed, maybe in a slightly more proactive way, and offer it in a much more proactive way as well. And to roll with the punches a bit more, there’ve been a few crunch points, but just not to take it personally.” *0103, Programme Director*  “I think, I don’t know, it depends on how resilient you are, but I’ve learned resilience because you know that the things you might be stressed about one day, probably aren’t going to be the things you’re stressed about the next day and the next day.” *0106, General Manager*  “I have coping mechanisms I’ve developed that put up glass screens in between people and me so that says I don’t take their stress. So, I’m very good in a crisis, I don’t take their stress and I teach people that resilience and how to manage what is going on around them at work because they need to do that and nobody teaches you that. You have to learn those techniques and what works and try something different and I do that extremely well to the level that people say you never get stressed.” *0107, General Manager* |
|  | Development of resilience | “Well, the more good people you appoint, the less sort of hands-on management you have to do. And then you can apportion things to different people, which I’m doing more of now, which makes things… That’s quite important from a resilience point of view.” *0094, General Manager*  “And if you’re not used to that, it can feel like you need to give everybody everything, but actually I always find that if you talk to people, try and get to the bottom of what the problem is, it normally pays off. But, you know, you get a bit of a thick skin; you have to because I don’t think they mean it personally, normally they’re fighting for their patients.” *0095, General Manager*  “there is a bit of a balance and I think my own instinct or my own self-preservation, I guess, I’m the type of person that when they ring up and say, can you come into my office, I automatically assume that I’ve done something wrong. Because if I start from a place where I’m in the wrong, then anything is going, because if I’m in the wrong, I’m already dealing with that. If it’s better than that, then it’s always better” *0096, General Manager*  “Yes, because you also find similar themes that crop up in a cycle every few years. If you’ve got three or four different services, it might happen in one service, and then you’ll see the pattern emerging in another. But because you’ve done it, experience is one thing. But just going back to that point is it is about patient care, but equally it’s about caring for the people who deliver that care too. Because that’s a key point, is that if you see something emerging with one team who’ve really had a bad time of dealing with whatever it was, you put steps in place to protect the next team that you can see that problem emerging.” *0099 Operations Manager*  “And more experience in terms of being able to deal with them to a certain extent. So, I wonder what the ten year ago me would have made of these last 12 months and some of the challenges we’ve had. But largely your experience is the more bruised on occasions you get, the better you should be in the future. As long as you take the learning from those. So, I’d like to think that there’s something around that ten years of experience that makes it more effective at communicating the downs as well as the ups” *0101, Hospital Director*  *“*I think, learning. Just learning from experience, really. The things that you might, you know, you get a hammering in one meeting about something, and then, by Friday, there’s something different to worry about, and no one’s remembered that. It’s things like that. It’s just learning how to deal with things. And also, I think, when you get hammered in a meeting, it’s usually because someone else has worried and stressed about something.” *0106, General Manager*  “Huge frustrations. I have learned to compartmentalise everything. That’s not only with the frustrations of not getting things done and the impotence of getting things done that is the NHS, but it’s also I compartmentalise everything that I do so that I don’t take work to other areas of my life and I don’t take other areas of my life to my work.” *0107, General Manager*  “I’ve become courageous on behalf of the people that I serve. I’ll probably put it that way. I would hope that in that courage, probably, I’ve had to learn the courage to be vulnerable as well. Because in terms of resilience, if you think of resilience in the fact that actually, you’re carrying a huge amount of information and worry, and input and output, and all of that. And, everybody’s stress and concern and anxiety, and timelines and tensions, and everything else. So, absolutely. Loads of it.” *0108, Divisional Director* |
|  | Contributors to low resilience | “It is really typical, when I think what we feel, quite often, myself, is stuck in the middle of. And, trying to manage the expectations from our staff, but also try and lobby those above us to try and get some change. It’s just really slow going. It’s really difficult” *0095, General Manager*  “And, actually, we’re here because we feel that we want to make a difference, and we’re here because we believe in it, and therefore, we’re not the people, necessarily at the frontline. We do get a lot of bad press, and it has been in the press, about how terrible managers are, and so much money is spent on managers. They’re a waste of time and resource.” *0105, General Manager*  “I think it’s quite lonely. I think it can be quite a lonely job, and that’s where you really need to seek your support from your triumvirate and the other peers. Because, although you can talk to your deputy and your direct reports about stuff, and I get on really well with mine, I can’t burden them.” *0106, General Manager*  “I think that’s something to watch, because that means your talent drain at level, and people will get burnt out. So, yes, that will be the piece I just wanted to share with you. I think it’s that kind of continuous piece that, and particularly at the moment, when it’s post-COVID too. I think it’s hard for people.” *0108, Divisional Director*  “I’m going to be really honest, it affects your resilience negatively, because you just get so sick of the waste of your time, I think.” *0116, General Manager*  “And I’d say the frustration impacts on your resilience, because you just think, am I going to ask my teams to go back and look at their processes to see if they can shave off £10,000 here or £5,000 there? The time it’ll spend for them to do that is probably more than the saving that they’re trying to make, that we have done this and we can’t think of anything else.” *0116, General Manager*  “As a service manager or business manager, you’re the one that gets the brunt of the clinicians’ frustrations.” *0117, Service Coordinator*  *“*I think the bit that’s tricky is accepting that not only do you not know what all the problems are, but you can’t fix all the problems and that’s the bit that becomes quite hard.” *0100, Divisional Director* |
|  | Provision of support for managers in comparison to clinical staff | “I think first of all, I think it’s been quite annoying that the government and the wider public sort of define the NHS as doctors and nurses. I mean, they don’t even talk about like, therapies and paramedics or any of that. So I think very often, the big national stuff is directed at those groups anyway. I think certainly with junior doctors and with nurses, it was pretty ropey before COVID, and COVIDs just pushed a lot of it over.” *0100, Divisional Director*  “Let’s call for this. We’ve had a change of director recently who’s very, you need to take care of yourself, think about you. Before that, I think that there wasn’t that focus. So I think things have changed direction for the better over the last two or three months.” *0102, Deputy General Manager*  “If you know you need support on something, it depends on what sort of person you are, but if you need someone to talk to, you’ve got to seek it out. No one’s going to deliver you a support package on a plate, we’re all big enough and grown up enough, I think, to try and be trusted to access that ourselves.” *0103, Programme Director*  “I don’t think we are. We absolutely not, but I think, even if it was offered to us, we wouldn’t accept it because there’s an exception. It’s not for us because our lives aren’t as difficult as theirs,” *0105, General Manager*  “I think, up to this point, there probably was not. Even thought, if you look in terms of response, there was not an adequate recognition of the impact it has on managers.” *0108, Divisional Director*  “No, not at all, not even a… Don’t get me wrong. From our line managers we get those, like, oh, well done, thank you so much. But nothing.” *0109, General Manager*  “When the trust is trying to make an attempt to save money, the first vacancies that would be cut are admin, so you think you have to put through a good case to say why you really need the staff that you need, whereas you probably wouldn’t get that challenge with a clinician or nurse or for someone with a clinical background, I think.” *0110, Business Manager*  “And like you said, we’re forgotten as admin I would say. That there’s this whole work we’re doing in the background, but nobody really thinks that we still have to come to work every day. We have to do all of that. We still have a personal life and problems in personal life…….. So, I think we are a little bit overlooked in how things can be done.” *0112 Service support manager*  “There is no forum for us other than each other in terms of peer review, peer-to-peer support. There is nothing for general managers where you can have that outlet.” *0116, General Manager*  “I think as a manager, you try and put on a brave face, because you’re also very aware that for your staff, you need to make it seem like everything’s fine.” *0117, Service Coordinator*  “I don’t know. I think I’ve never really, I suppose, asked for that help. I think we are expected to just get on with things sometimes, and I think there are managers who… I’d say for me, I suppose I’ve been around a few years, but younger managers, I can imagine, would find that really upsetting and stressful. It probably is something that could be done a bit better, I think, in terms of that, because you do get… As a service manager or business manager, you’re the one that gets the brunt of the clinicians’ frustrations.” *0117, Service support manager.*  “And we just keep going and going and going.” *0102, Deputy GM* |
|  | Ability to say if not coping | “Not only do not think managers have had that level of influence, but I think managers are bad at saying they need it…… So no, I don’t think we’ve been very good at it, but I think that’s a profession, I don’t think we’ve sorted it out. I think we’ve just done the whole humble, British, just soldier on.” *0100, Divisional Director*  “I think we support each other, and sometimes, you know, it’s not PC for a manager to say I’m struggling, whereas it might be for a clinician.” *0104, General Manager*  “Oh, absolutely. You can’t admit that you’re struggling. You have to do long hours. It’s accepted of you. You can’t possibly leave at five o’ clock. And, definitely, most people are martyrs, of course. But they might think that’s… It’s again, because it doesn’t pay that well, so it’s kind of, really values-driven.” *0105, General Manager*  “But I think, to say, do you know what, I’m really not coping here. I think, yes, that would be difficult to admit….. Because you’re at the top. You want to become this GM. It’s quite hard. You get there and then, suddenly, to say, actually, do you know what. You know, I think it would be hard. I think it would be hard to admit.” *0106, General Manager*  “I think you’re just expected to just get on and do it, if I’m honest, and there is an element of resiliency you just need to have.: *0110, Business Manager*  “Just get on with it. This is contained within your directorate. It doesn’t matter what happens if you’re the accountable person it’s going to come back to you. There’s always a bit of a hesitance of people to take annual leave because you can’t cancel clinics, you can’t get someone on banks to cover your ward shift. It just is an ongoing thing. So, there is no allowance on those limited things” *0113, General Manager*  “Sometimes, you could work all hours under the sun and nobody would ever say, no, stop and go home and have a rest, they would just be like, well, that’s fine. There is an expectation, I think, as a manager that you are on-call all the time as well.” *0117, Service Coordinator* . |
|  | Presence of peer support | *“And we actually found that we found a voice as a group, and then our CLO took that on to chair the group and really listen to what people say. And it was quite interesting, because if you get all of us together, there’s actually not a lot that we can’t do. And there’s not a lot of people that can say we can’t do it, either, because we’re all together.” 0094, General Manager*  *“I think at best we used to come together once a week for about an hour with a very set agenda. And the aim was just to get through the agenda and clear off again. As opposed to actually use the time effectively to share ideas, to share skills, to share expertise.” 0101, Hospital Director*  *“I think particularly within the divisions, so the medicine peer group, I can see evidence of people sharing ideas and being quite collaborative, there’s times more or times less.” 0103, Programme Manager*  *“Yes. I think for me, the most important relationship you can have is with your peers. Your GM colleagues. I think one of the things that’s really been lacking, you don’t really get that in lockdown, and all the distancing. You don’t have that. I think, at these senior levels, a supportive boss can be good, but ultimately, they need to get on with the job. At this senior level, you’re expected to do it and get on with it.” 0106, General Manager*  *“I’m not sure about that, actually. I don’t think so. I think it’s more about learning from what you’re, you know, this is what [unclear]. Oh, we’ve got this set target. How are you going to do it? What you doing? That is the most important relationship for me. That it’s not like point scoring. It’s like a collaborative team work. There’s always some people there who want to be the best, and so, don’t share information. Knowledge is power. Or, I know something you don’t know. But I think most of the GM groups are really collaborative.” 0106, General Manager*  *“And then we are trying to change the direction the trust is going and to influence decisions. If you think, there are only 12 of us, and between 12 of us, we are managing this hospital completely.” 0109, General Manager*  *“And they will call me and say, oh, because I’m really good in operational management, I’m very good with recoveries and things, so they would ask my advice. I recovered a couple of our problematic services. We do that. We’re quite a good group now, I think.” 0109, General Manager*  *“I think some sort of more formal, structured training at the outset, I think would be a good thing. I think the peer to peer, enabling some sort of more formal peer to peer support, I think is good. Yes, I think those would be the two things that would make a difference.” 0111, Business Manager* |
| **STRESS** | Accountability and potential repercussions | “I am the accountable officer for it. So, I suppose, it’s as much me. Yes, pluses or minuses come down to me in the end, so obviously the management teams or even some of the nursing teams, particularly, will have a responsibility around the management of money. So, for ordering and staffing and all of those things that make up the spend. But, ultimately, what we do or don’t do and how we perform, is accountable to the general manager, yes.” *0096 General Manager*  “Because we’re supposed to know. We’re the ones who fix everything. So when we’re not in a position to be able to fix it, and we’re just waiting for the next instruction, you’re almost negligent. That’s how it feels, or that’s how it is viewed, that you’re being negligent. You’re not looking after us.” *0099, Operations Manager*  “I’m not sort of like, going into my performance review with my boss, I’m going to balance all my books. I’m more like, I’m going to make this look as good as possible, because the money’s bad. And the only way I could fix it is broad brush changes to services which would be in no one’s benefit. So it would be saying, I know you’ve got these gaps on your anaesthetic rota that you can’t appoint any more consultants, because I want to save that 200 grand you’ve got. And you’re just going to have to make this work.” *0100, Divisional Director*  “Or if we do this how do we manage because the finance don’t have a finite amount of money there, how do we manage the whole thing to say, we can do it within budget or the consequence of not doing it is such that we need to do it and just accept that finances are going to be shocked if you like.” *0107, General Manager*  “You take it in the next year. Not for me personally. This is one thing, downfall [?], with the NHS, is that there is not a lot of accountability. I think there should be, but I think because they’re giving us such unreasonable targets sometimes, accountability is lacking, because they know we can’t meet it. But, e.g., I met every single target, CIP target, I ever had, and what I get in return is certain business cases approved that potentially would not be approved if I did not meet my standards, if that makes sense.” *0109, General Manager*  “I don’t think so, but then at the same time, I think it’s probably a conversation, that it’s probably the GMs who sit in front of the finance board of directors to explain what’s happened there…… And I think that’s probably the bit, is when you’re… I think the most stressful bit is having to sit in front of a panel of people and explain why you weren’t able to deliver the impossible.” *0110, Business Manager*  “So it depends now, it’s different than obviously, previously if you breached, 52-week breach, for example, for treatment, which in our case it’s a surgery, it was a financial impact on the department. NHS England has you then on their list that you have officially too many breaches and your managers asking, why did that happen? And then you have investigations and all of that.” *0112, Service support manager*  “I would say the ultimate source of stress is always there are lots of people who are given designated roles and paid for certain roles could be [unclear] roles, audit roles, quality roles, whatever. But if it’s ever not done, it always goes back to the business manager or general manager There’s always a deep lack of accountability elsewhere. So even though it may not be the managers role, they will be dragged in to fix it at last minute under huge stress.” *0113, General Manager*  “Even though we work as equals, at the end of the day, if anything goes wrong, then all the joy of the accountability sits with me.” *0116, General Manager*  “Oh, you get monthly… Terrible show-and-tell meetings, so they’re performance reviews. You have to turn up with your, what are they called? I can’t remember. It’s basically when you’re on the naughty step, what are your measures to bring it back in line and what are you doing? You have to produce yet another set of countermeasures, that’s it, against your poor performance against your SIP” *0116, General Manager*  “my job is to make sure that we demonstrate we’re meeting those targets or standards, because if you don’t there will be some financial impact on us.” *0109, General Manager* |
|  | Bureaucracy | “I think my frustrations as a manager and as part of that triumvirate is very much there’s a lot of obstacles/hoops to go through.” *0101, Hospital Director*  “It’s painful. We know our service better than anyone. We know we need more consultants. We know that consultant’s left, so you have to replace. So why do I have to go and tell someone who’s sitting on an executive board that ITU needs to replace a consultant that’s left. Because there won’t be enough consultants then. I think if it was a new post, I understand why that has to be agreed. But for things that are like for like replacements, I’m not too sure why someone that’s sitting on a board makes that decision for me.” *0102, Deputy General Manager*  “The difficulty for me is, again dipping on the external interface, is that my finances and my director of influence are completely bound up with other organisations. So, my ability to say change the discharge function. I’ve got to consult in quite a complex web with lots of other external organisations that have their own budgets, and their own drivers, and their own pressures, and their own staffing concerns, and their own HR processes, that’s extremely complicated. But it’s not impossible.” *0103, Programme Director*  “And I think, for such a large organisation, it’s so bureaucratic, and everyone is after the same pot of money, and the same slice of the pie, if you were.” *0105, General Manager*  “But then anything that we do do takes forever to be resolved. So there are things, there are business pieces I have put in back in August last year that I am only now, and we’re nearly in August, so it’s 11 months. It’s a long time pacing August to now in May. So, we’re only now starting to chip away and get some of the items resolved so I have part of my service that I need to develop.” *0107, General Manager*  “I think there is far more bureaucracy in the NHS that impacts on what we do. So, the red tape to get things done and the delays that are out in about decision making really makes the job hard.” *0107, General Manager*  “I think, as a trust, it can be a bit like walking through treacle in finding out how to do things. And even though I’ve been in the post for about five years, there are still lots of things that I don’t know how to do. At least now, I can normally know who I should speak to, who should be able to help me, but the systems in the hospital, they’re not transparent, and it can take a long time” *0111, Business Manager*  “But that involves having to put a case together to present to higher people in the management chain to say this is why we need an extra dietician, because we need to see more people and we need to give them more support and we need to be able to put on more dietetic clinics. But making that case is quite difficult and those business cases are really difficult to get through.” *0117, Service Coordinator* |
|  | Expectations and targets | “And although we always say to people, we don’t expect you to reply all the time, I think a lot of more junior people feel that they should be checking their emails even on their days off and things” *0094, General Manager*  “I think that we probably have quite a lot of unrealistic expectations put on us. So, just to qualify that, I suppose that stress in your role is completely dependent on what you’re doing maybe.” *0098, General Manager*  “I think we’re responsible for an awful lot that we don’t have control over, and there’s a bit about, it’s just the job. But that can also be really frustrating, because you think to yourself, why am I getting crap about this thing that I had no idea was happening.” *0100 Divisional Director*  “And sometimes the speed of response is totally unrealistic. But once again, I think that’s part of the job and it’s part of the political agenda that you’re working with.” *0101, Hospital Director*  “To try and explain to them how slow-moving things are, and that you’ve had a really difficult shift, but there is no more money and we have to make do. It’s hard, because you’re the person that has to deliver those messages, and I don’t necessarily agree with them. You just toe the party line. It’s hard.” *0105, General Manager*  “And it’s like everything needs to be done yesterday.” *0110, Business Manager*  “Definitely, because we’re given a budget and then we’re told to find 5% savings, and there’s a pressure on you to try and be imaginative” *0116, General Manager*  “Or you’ve got a big project and you’re dependant on other people actually. Sometimes that’s frustrating because they’re not working to your timescales; they’re working to very different timescales, as such.” *0104, General Manager*  “And periodically, weekly, monthly, we’ll have our deliverables that we are measured against. We present those. We make sure those are working to the governed requirements.” *0099, Operations Manager*  “So, it’s a great service to offer, but in order to keep up with the demand, which is just growing and growing and growing, we’re actually constantly managing our resources. So, my role is around ensuring that we hit all the access targets.” *0111, Business Manager*  “And clearly delivering on all those ideas in a financially difficult environment means you are always going to have some people feeling disappointed around what has and hasn’t been delivered. I think my view, as long as we are honest in terms of the reason for not being able to deliver on something and the rationales for not being able to deliver on something.” *0101, Hospital Director* |
|  | Responsibilities | “But most of the time it’s genuine, you know, if someone needs investment in their services, then my job is to try and help them get that I think” *0095, General Manager*  “I’m responsible for the day to day sort of operational running of all the services and the money, the HR, the planning, the performance, the emergency response. Pretty much everything apart from, in terms of area of actual accountability, like the clinical governance. And everything else sits with us. Although broadly you would say that we’re still responsible for governance and [unclear] everyone responsible for governance. And like quality and safety sits underneath all the other stuff we do.” *0100 Divisional Director*  “And I still feel quite stressed by the responsibility of that and making sure they’re okay. Even though I know it was the right thing to do and I stand by the decisions I made” *0103, Programme Director*  “But, you know, it’s a really high focus on quality and safety. Just the smallest thing we do could have such a significant impact. That in itself, and trying to manage that, across three sites and so many beds, and so many staff.” *0105, General Manager.*  “I think it’s the weight of the responsibility. I know, some of the times when I’ve had sleepless nights, it is around some of the things that we deal with. And I’ve often had this conversation with consultants. I’m not saying it’s any less difficult, absolutely. I’m not saying, what we do is more important…..And, it’s carrying that burden of responsibility when it isn’t legally, you are… There’s lots of these pockets of things happening, and if you don’t have a responsive senior team and then it stops with you, and you’re trying to get people to understand that that’s a risk and that’s important.” *0105, General Manager*  “When someone asks me, what do you do, it’s like everything. Basically, the accounts board officer for the directorate. Financially, in terms of governance. So, part of a triumvirate who leads the directorate.” *0106, General Manager*  “Particularly troubleshooting, particularly there is a problem we need to solve this. You have to very much think on your feet, very much run when you might not know how to run, very much trying to find solutions because we can’t just say it can’t happen. It’s about finding a solution and thinking, I need to make something happen, where do I get the funds from? And being that interface between the financial side, the people on the ground who just want to do their job and the making sure everything is safe and secure and the patients safety is managed correctly.” *0107, General Manager*  “You’re responsible for everything from, in terms of quality of care, performance management, operational, plus finance, and the workforce piece.” *0108, Divisional Director*  “Also, every single incident, any serious incidents, any recruitment issues, any big HR problems, any HR issues, that’s all within my remit. It’s quite a massive remit, basically making sure that all my directorate is working every day.” *0109, General Manager*  “But I do treat it like it’s my own money. Because I think if you don’t, or certainly for me, if I don’t, it’s just so much, it’s so big, and there’s so much of it, that you just think, well, I just don’t really know, let anything happen that’s going to happen.” *0096, General Manager*  “Because I see when people are budget setting, or business planning, they never factor in what is it that’s needed. What is it that’s needed, what resource do we need to support the doctors and nurses. We don’t do that, we just go how many more doctors, and how many more nurses do we need. So I think that causes a lot of stress.” *0102, Deputy General Manager*  “If we are over budget so we don’t have the budget to spend then there is going to be that challenge that says, I need to do something for patient safety. And then the finance are going to kick in and say, you can’t do it because you don’t have money. And then there is that discussion on how important it is against all the other conflicts that are out there. And there’s a decision making process we need to go through that says if we do this we can’t do that.” *0107, General Manager*  “So, if you’re looking at individuals who have to manage businesses that turn over £50 million a year that’s a big-time business, and that’s a big-time team, but that’s similar to what each General Manager at [Company] do. And it’s like a big-time business. You’ve got all of your huge expensive equipment, you’ve got all your expensive clinicians, you’ve got your safety metrics, quality metrics, financial accountability. That’s a full business unit within a wider hospital” *0113, General Manager* |
|  | Workload | “So we end up with these very hectic weeks where you work eight till six, eight till seven, pretty much every day, you do a couple of hours over the weekend.” *0100, Divisional Director*  *“*But that is the job and you just end up working up a lot of hours and you’re tired and frustrated that you can’t fix everything. And in moments of being rational, because a rational human being goes, it’s impossible to fix everything and he’s not worried about it. When you’re not being rational because you’re tired and because you’re being pushed, that it itself is quite stressful” *0100, Divisonal Director*  “because unfortunately everyone comes to you, but you don’t have anyone to go to. And then it gets to the point where you’re not sleeping. Managers don’t get paid over time. I would be going in on the weekend. You don’t get paid for that. That’s my decision to go in.” *0102, Deputy General Manager*  “Generally, yes, although sometimes you think that, you know, sometimes you think, oh, my God, it’ll be nice just to do your shift and go home whereas in your operational role you tend to work in the evenings, into weekends, etc.” *0104, General Manager*  “would say when I first came to [Company] I would do 20 hours of unpaid time a week just continuously. It’s down a bit now down, it’s down to about ten or 12. But it’s more just the hidden thing and more jobs and things are given and you just either have to fit it in or work later. That is a common culture in everywhere I’ve been managers will work very, very late for no time back” *0113, General Manager*  “Because the expectation is, if you are a general manager, you have 37 and a half hours in your job description, but really everyone works 50. Why are you complaining because you’re working five ten-hour days…..My boss has even said to me, that’s the NHS, that’s what you expect, GM, you should be working 50 hours a week because that’s how busy you are. Anything above that, this is what’s expected of you.” *0116, General Manager*  “It’s really difficult to know who to actually focus your attention on. So you try and learn from everybody equally, but principally there will be areas that have more problems than others. Or at least there are areas that will be more vocal about the problems that they’ve got” *0100 Divisional Director* |
